# Combined vitamins D3 and K2 as potential modulators of multiple myeloma cell pathophysiology: transcriptomic and epigenetic effects under standard culture and bone marrow-like conditions

**DOI:** 10.3389/fonc.2026.1940737

**Published:** 2026-09-14

**Authors:** Karolina Łuczkowska, Patrycja Stodolak, Piotr Kulig, Bogusław Machaliński

**Affiliations:** 1 Department of General Pathology, Pomeranian Medical University, Szczecin, Poland; 2 Pharmaceutical Facility of Pomeranian Medical University, Szczecin, Poland; 3 Department of Hematology and Transplantology, Pomeranian Medical University, Szczecin, Poland

**Keywords:** DNA methylation, hypoxia, multiple myeloma, RNAseq, vitamin D, vitamin K

## Abstract

**Introduction:**

Multiple myeloma is an incurable hematological malignancy that requires extensive research to understand its biology and identify novel therapeutic strategies. Hypoxia within the tumor microenvironment affects numerous biological processes, including enhancing cellular stress resistance, promoting angiogenesis, shifting metabolism toward anaerobic glycolysis, and modulating gene expression and epigenetic modifications.

**Methods:**

In this study, we investigated the effects of combined vitamin D3 and vitamin K2 treatment on MM cells cultured under standard and bone marrow-like conditions, emphasizing the critical role of oxygen levels in cell culture. Transcriptomic (mRNA sequencing) and DNA methylation analyses were performed exclusively for the combined vitamin D3 and vitamin K2 treatment to characterize the molecular changes associated with this intervention.

**Results:**

Transcriptomic and DNA methylation analyses comparing cells treated with the combined vitamin D3 and vitamin K2 regimen with untreated controls (adjusted p<0.05) identified 16 differentially expressed genes (DEGs) and 83 differentially methylated positions (DMPs) in U266 cells, and 4,441 DEGs and 26 DMPs in MM1S cells under bone marrow-like conditions. Under normoxic conditions, 444 DEGs and 5,269 DMPs were identified in U266 cells, whereas 4,575 DEGs and 8,710 DMPs were detected in MM1S cells. These findings demonstrate that the molecular response to the combined vitamin treatment differed between MM cell lines and oxygen conditions, with a particularly restricted transcriptional response in U266 cells and reduced DNA methylation changes under hypoxia. These findings suggest cell line-specific adaptation mechanisms to low oxygen conditions, similar to those occurring in the tumor microenvironment in vivo. Focusing on the effects of vitamin treatment under hypoxia, we identified several genes, including ABCA1, ADAMTS9, and RUNX1T1, that may contribute to the antitumor mechanisms of VD and VK. Notably, hypermethylation of the MCOLN3 gene correlated with reduced expression, identifying MCOLN3 as a potential target for further investigation into the molecular effects of vitamin D3 and vitamin K2.

**Discussion:**

Overall, these findings demonstrate that oxygen availability influences the antitumor effects of combined vitamin D3 and vitamin K2 treatment and reveal molecular changes that may contribute to their mechanism of action. They also highlight the importance of considering physiological oxygen conditions in in vitro studies of multiple myeloma.

## 1 Introduction

Multiple myeloma (MM) is an incurable hematologic malignancy derived from the malignant plasma cells of MM residing in the bone marrow. It is classified as a chronic and progressive disease, characterized by alternating periods of remission and relapse, with clinical and molecular pathogenesis marked by substantial heterogeneity (1–4). Despite the introduction of novel therapies, including proteasome inhibitors, immunomodulatory drugs, and monoclonal antibodies, MM remains a disease with a high risk of relapse and resistance to treatment. Therefore, there is an urgent need to explore new therapeutic strategies aimed at modulating the intracellular survival pathways of MM cells and their interactions with the bone marrow microenvironment (2).

The molecular basis of MM encompasses both genetic and epigenetic alterations that jointly drive malignant transformation, disease progression, and drug resistance. The most common cytogenetic abnormalities include translocations involving the *IGH* region (14q32), *RB1* deletions, 1q gain, hyperdiploidy, and mutations in *NRAS*, *KRAS*, and *TP53* (8, 9). Particularly unfavorable prognostic factors are represented by translocations t (4,14)(p16;q32), t (14,16), t (14,20), chromosome 1 abnormalities, and 17p deletion involving the *TP53* gene (5–8). Alongside these alterations, increasing attention has been directed toward epigenetic dysregulation, which significantly shapes disease biology and may represent a promising therapeutic target (9–11).

An essential factor in the pathophysiology of MM is the bone marrow microenvironment, where malignant cells reside and adapt to local conditions. Of particular importance is hypoxia, a physiological hallmark of the hematopoietic niche. By regulating bone marrow cell function, hypoxia promotes metabolic changes, enhanced proliferation, survival of malignant plasma cells, and epigenetic modifications (12–14). Stabilization of the transcription factor HIF-1α triggers adaptive molecular pathways while simultaneously inducing epigenomic remodeling, further contributing to the complexity of disease progression. It is noteworthy that most *in vitro* studies are conducted under normoxic conditions (21% O_2_), which fail to accurately reflect the hypoxic environment of the bone marrow (15–18). Because hypoxia profoundly influences gene expression, epigenetic regulation, and intracellular signaling pathways, it may also modify the cellular response to compounds with regulatory activity, including vitamins with potential antitumor properties.

Metabolic and environmental factors, such as vitamins with potential modulatory activity, also deserve particular attention. Vitamin D (VD), a prohormone activated in the liver and kidneys, exerts its effects via the nuclear receptor VDR, regulating the expression of numerous genes (19). Although traditionally associated with calcium–phosphate homeostasis and bone metabolism, an increasing number of studies highlight its pleiotropic, so-called nonclassical actions (20). These include the regulation of immune responses and modulation of tumor-related processes. VD deficiency, frequently observed in MM patients, is associated with poor prognosis and higher mortality (21–23). Studies have shown that VD can inhibit cell viability, induce differentiation, and influence the epigenome by modulating DNA methylation, histone acetylation, and microRNA expression (24–26).

Vitamin K (VK), particularly in the form of menaquinone-7 (MK-7), has also attracted growing interest. Beyond its well-established role in blood coagulation and bone metabolism, VK exhibits antitumor properties, including the induction of apoptosis, inhibition of proliferation, and epigenomic regulation through promoter methylation and histone acetylation (27–31). Among different VK isoforms, MK-7 is characterized by high bioavailability and a longer half-life, which may contribute to its sustained biological activity. Previous studies have indicated that VK derivatives may inhibit tumor cell growth and induce apoptosis in several cancer models. However, the biological effects of VK/MK-7 in multiple myeloma remain insufficiently explored, and its potential role in regulating malignant plasma cell biology requires further investigation. Although its antitumor effects are less extensively documented compared to VD, accumulating evidence suggests that the combined activity of both vitamins may exert synergistic effects. In clinical practice, vitamin K2 is frequently considered as a complementary supplementation to vitamin D3, particularly due to the synergistic roles of both vitamins in calcium homeostasis and bone metabolism. This combination may be of particular relevance in multiple myeloma patients, who are at increased risk of skeletal complications due to disease-associated bone remodeling abnormalities. This synergy, well established in bone and cardiovascular physiology, may also contribute to the modulation of molecular and epigenetic plasticity in malignant cells. Considering the complementary biological functions of both vitamins, combined VD and VK treatment may represent a promising approach for modulating malignant plasma cell behavior and the bone marrow microenvironment.

To the best of our knowledge, this is the first study to investigate the effects of combined vitamin D3 and vitamin K2 treatment in multiple myeloma cells under both standard and bone marrow-like conditions, providing new insights into how oxygen availability influences the molecular and biological responses to vitamin-based therapy. The primary aim of this study was to investigate the effects of combined vitamin D3 and vitamin K2 treatment on multiple myeloma cells under standard and bone marrow-like conditions and to determine whether oxygen availability influences the biological response to vitamin treatment. The secondary aims were to: (i) characterize transcriptomic and DNA methylation changes associated with combined vitamin D3 and vitamin K2 treatment; (ii) identify molecular pathways and candidate genes potentially involved in the antitumor effects of vitamin treatment; and (iii) evaluate the importance of applying physiologically relevant oxygen conditions in *in vitro* studies of multiple myeloma. We hypothesized that combined vitamin D3 and vitamin K2 treatment would exert antitumor effects on MM cells and that the magnitude and molecular characteristics of this response would be modulated by oxygen availability.

## 2 Materials and methods

### 2.1 Cell culture general course of the experiment

In this study, the U266 and MM1S human multiple myeloma cell lines (ATCC, Manassas, VA, USA) were utilized. The cells were maintained in RPMI-1640 medium (ATCC, cat. no. 30-2001), supplemented with 2 mM L-glutamine, 10 mM HEPES, 1 mM sodium pyruvate, 4500 mg/L glucose, and 1500 mg/L sodium bicarbonate, following the manufacturer’s guidelines. To create a complete growth medium, fetal bovine serum was added to reach a final concentration of 15%. The culture medium was refreshed every three days. The concentrations of vitamins 25(OH)D (Sigma-Aldrich, St. Louis, MO, USA) and K2MK-7 (Sigma-Aldrich) were determined experimentally using the Alamar Blue assay (Thermo Fisher Scientific, Waltham, MA, USA), and the results are presented in subsection 3.1. After testing various concentrations, myeloma cells were incubated for 48 hours with the selected doses of 10^-6^M 25(OH)D and 2.5×10^-7^M K2MK-7 for subsequent experiments. In addition, the selected dose of vitamins is not toxic to fibroblasts (see subsection 3.1). This approach was adopted to ensure that the selected doses exerted biological effects on malignant cells while remaining non-toxic to non-malignant cells, thereby reflecting concentrations with greater potential translational and clinical relevance rather than maximizing cytotoxicity. Cell cultures were maintained both under standard conditions (~14.5 PSI and 21% O2) and under conditions similar to the natural environment in which multiple myeloma occurs, i.e., bone marrow-like low-pressure/hypoxic conditions (1 PSI and 6% O2). These parameters were selected to mimic the physiological environment of the bone marrow, where multiple myeloma cells reside. To establish bone marrow-like conditions, cells were cultured under reduced oxygen and pressure conditions using an Avatar system (Xcell Biosciences; San Francisco, CA, USA). The study consisted of the following research groups: a) myeloma cells incubated with 25(OH)D; b) myeloma cells incubated with K2MK-7; c) myeloma cells incubated simultaneously with 25(OH)D and K2MK-7. The control group consisted of myeloma cells not treated with vitamins. Both vitamins were initially dissolved in ethanol and subsequently diluted in culture medium to the desired working concentrations. A vehicle control containing the corresponding concentration of ethanol (maximum final concentration: 0.01% v/v) was included in all experiments to exclude potential solvent-related effects. Cells from each research group and the control group were assessed for cell viability. However, for mRNA-seq and methylation array analysis, only the control group and the group simultaneously treated with 25(OH)D and K2MK-7 were selected. The 25(OH)D form was chosen for this study because it is significantly more chemically stable than the 1,25(OH)2D form, which has a very short half-life and is easily degraded. This short half-life, combined with possible catabolic mechanisms (e.g., via the CYP24A1 enzyme found in U266 and MM1S cells), may limit its sustained activity in cell cultures. The 25(OH)D form appears to be more suitable for obtaining reproducible and reliable results in cell cultures.

### 2.2 Assessment of cell viability over time

Cell viability over time was assessed using the Alamar Blue assay (Thermo Fisher Scientific). The study aimed to demonstrate the long-term effects of a single incubation with vitamins on myeloma cells. The experiment was performed in three biological replicates for each cell line, with six technical replicates per biological replicate. Therefore, cells after 48 hours of vitamin incubation and a control group were seeded into 96-well plates (15000 cells/well). Changes in cell viability were assessed on days 1, 3, 6, and 8 of culture.

### 2.3 DNA extraction and bisulfate conversion

Genomic DNA was extracted from three independent cell culture replicates for each experimental group using the PureLink Genomic DNA Mini Kit (Thermo Fisher Scientific), following the protocol provided by the manufacturer. DNA yield and integrity were evaluated with the TapeStation 4510 system (Agilent Technologies, Santa Clara, CA, USA) using the Genomic DNA ScreenTape kit (Agilent Technologies). All DNA samples exhibited a DNA Integrity Number (DIN) of 9 or higher, indicating excellent quality. For bisulfite conversion, 500 ng of DNA per sample was processed using the EZ DNA Methylation-Gold Kit (Zymo Research, Irvine, CA, USA) according to the supplier’s instructions.

### 2.4 RNA isolation

Total RNA was isolated from three biological replicates per group, each derived from 1 × 10^6^ cells. The extraction was carried out using the mirVana miRNA Isolation Kit (Thermo Fisher Scientific) in accordance with the manufacturer’s instructions. RNA quantity and integrity were evaluated using the TapeStation 4510 system in combination with the RNA ScreenTape kit (Agilent Technologies). All samples achieved RNA Integrity Numbers (RINs) of 9 or higher, indicating excellent RNA quality.

### 2.5 Methylation arrays

DNA methylation profiling was performed using the Infinium MethylationEPIC v2.0 BeadChip Kit (Illumina, San Diego, CA, USA). Each experimental group included three independent biological replicates (n=3). After bisulfite treatment, DNA was amplified for 24 hours, enzymatically fragmented, and precipitated to prepare it for hybridization. Samples were hybridized to the BeadChip at 48 C for 20 hours. Following hybridization, non-specifically bound DNA was removed through washing steps. Primer extension was then carried out using labeled nucleotides, and the arrays were stained. Final detection was conducted on a NextSeq 550 system (Illumina).

### 2.6 mRNAseq

Sequencing libraries were prepared using the Illumina Stranded mRNA Prep Kit and IDT for Illumina RNA UD Indexes Set A (Illumina), following the manufacturer’s protocol. Each experimental group included three independent biological replicates (n=3). For each sample, 1000 ng of total RNA was used. RNA was first purified using oligo(dT) magnetic beads and fragmented for cDNA synthesis. Reverse transcription was performed with Actinomycin D to enhance strand specificity by inhibiting DNA-dependent synthesis. The second strand was then synthesized, followed by A-tailing and adapter ligation. Libraries were purified with the AMPure XP system (Beckman Coulter, Brea, CA, USA), indexed, and amplified to form clusters during sequencing (10 PCR cycles: 98 °C for 10 s, 60 °C for 30 s, 72 °C for 30 s). After a second purification, library quality and concentration were assessed using the TapeStation system and D1000 ScreenTape (Agilent Technologies). Libraries were diluted to 1.3 nM, pooled, denatured with 0.2 N NaOH, and sequenced at 1.3 pM on a NextSeq 550 (Illumina) platform using the High Output Kit v2.5 (150 cycles, Illumina).

### 2.7 Analysis of vitamin D receptor expression in U266 and MM1S cells

#### 2.7.1 Western blot

VDR protein levels were assessed using automated Western blot analysis. Cell lysates from the U266 and MM1S lines were obtained from three biological replicates and assayed using the Bradford method. The analysis was performed using the 12–230 kDa Fluorescence Separation Module (Bio-techne), EZ Standard Pack 1 12–230 kDa (Bio-techne), primary anti-VDR rat monoclonal (9A7) (Thermo Fisher Scientific) at a concentration of 1:20, and anti-GAPDH mouse monoclonal, Invitrogen GAPDH monoclonal anti-mouse (1D4) (Thermo Fisher Scientific) at a concentration of 1:20; appropriate secondary antibody anti-rat (Thermo Fisher Scientific), anti-mouse secondary antibody (Bio-techne)) and at a concentration of 1:200. The amount of protein used per capillary was 0.62 mg/mL. Samples were denatured according to the manufacturer’s instructions before analysis. The separation1 and analysis ware performed using a Jess apparatus (Bio-techne). Densitometric analysis of western blot data was performed using the dedicated Compass for Simple Western software associated with the Jess system. Integrated optical density (IOD-area under the peak) values were obtained automatically by the software, with background corrected and target protein quantification normalized to GAPDH.

#### 2.7.2 qRT-PCR

The qRT-PCR reaction was carried out in two stages: 1) reverse transcription of 0.1 µg mRNA using the First Strand cDNA Synthesis Kit (Thermo Fisher Scientific), followed by 2) the qRT-PCR amplification step. For each sample, the reaction mixture contained 5 μL SYBR Green PCR Master Mix (Bio-Rad, Hercules, CA, USA), 1 μL of cDNA template, 1.8 μL of specific primers (0.4 μL forward and 0.4 μL reverse), and 3.2 μL of nuclease-free water. To validate the constitutive gene and obtain the most reliable results, two genes, beta-2-microglobulin (*BMG*) and glyceraldehyde-3-phosphate dehydrogenase (*GAPDH)*, were used. The PCR reactions were carried out using a Bio-Rad CFX96 Real-Time PCR Detection System (Bio-Rad Inc., Hercules, CA, USA). Primers were designed with BLAST PRIMER and synthesized by the Laboratory of DNA Sequencing and Oligonucleotide Synthesis, Institute of Biochemistry and Biophysics, Polish Academy of Sciences, Warsaw, Poland. The primers used had the following sequences: *BMG* F 5’-AATGCGGCATCTTCAAACCT-3’; R: 5’-TGACTTTGTCACAGCCCAAGA-3’; *GAPDH* F: 5’-GAGTCCACTGGCGTCTTCAC -3’, R: 5’-GGTTCACACCCATGACGAAC-3’, *VDR* F: 5’ GACCTCACAGAAGAGCACCC 3’, R: 5’-CGTTCCGGTCAAAGTCTCCA-3’.

### 2.8 Bioinformatic analysis

Sequencing reads were demultiplexed using Illumina bcl2fastq (v2.20) to generate FASTQ files. Quality control was performed with FastQC, and Cutadapt (32) was used to trim adapters and low-quality reads. Processed reads were aligned to the human reference genome (hg19) using STAR (v2.5.2b) (33) with the corresponding GTF (34) file from Ensembl. Gene-level counts were obtained using featureCounts (35) and imported into R for TPM normalization. Genes with low median expression were excluded, and differentially expressed genes were defined as those with a fold change >2 or <−2 between study and control groups. To identify functional biological pathways associated with observed gene expression changes, enrichment analysis was performed using two complementary approaches: Gene Set Enrichment Analysis (GSEA) and pathfindR. GSEA was used to assess which gene sets are statistically enriched across the entire ranked list of genes, applying a significance threshold of *padj.* < 0.05. In contrast, pathfindR was employed to identify active subnetworks within the protein–protein interaction (PPI) network among statistically significant (*padj.* < 0.05) genes, enabling detection of biologically related gene clusters and their associated pathways. Combining these approaches provided both a global overview of potentially enriched pathways and detailed insights into gene interactions and modules relevant to the biological context under study.

Raw data were generated on Infinium EPICv2 bead arrays, and a corresponding CSV sample sheet was prepared to map each sample to its respective group. All IDAT files and the sample sheet were placed in a single directory in preparation for downstream processing in R (v.4.4) with relevant libraries. Quality assessment and preliminary data handling were carried out using methods available in the minfi package (36, 37). First, the RGChannelSet was created by reading the IDAT files, and metadata were retrieved via phenotypic data extraction from the sample sheet. Background correction and normalization were then executed with the Noob procedure, which helps to correct potential technical artifacts and variability in the fluorescence signals (38). The EPICv2 annotation was assigned to ensure accurate probe localization, and suboptimal probes were removed based on standard criteria to preserve data integrity. Once a MethylSet containing beta-values was obtained, additional QC filtering was performed with ChAMP, which enabled comprehensive analyses and facilitated the detection and removal of potential outliers or artifacts (39). Batch-associated variation was subsequently addressed via ComBat, which normalizes data across experimental batches (40). Beta-values were first converted to M-values, and batch correction was performed on M-values, as recommended for statistical analyses of DNA methylation data (41). The data were then reconverted to beta-values. Quality metrics were rechecked post-ComBat to confirm that normalization and adjustment did not introduce new biases. Differentially methylated positions (DMPs) were identified for each pairwise contrast, and an appropriate false discovery rate (FDR) threshold was set to control for multiple testing. A variety of visualization approaches were implemented, including Manhattan-like plots (with CpGs ranked by effect size), donut charts illustrating proportions of hyper- versus hypomethylated CpGs, and bar plots summarizing distributions. Annotations were conducted by referencing the EnsDb.Hsapiens.v75 database (42), enabling the identification of implicated genes. Lists of significantly hypermethylated and hypomethylated genes were subsequently generated for functional enrichment. Gene Ontology (GO) analyses were then conducted via the clusterProfiler package (43). Separate hypermethylated and hypomethylated gene lists were mapped to Entrez identifiers and subjected to over-representation analysis (ORA) for sets of hypermethylated and hypomethylated genes, and GSEA. For the GSEA, beta-based or M-based metrics were converted into a ranked score and ordered. Separate hypermethylated and hypomethylated gene lists were mapped to Entrez identifiers and subjected to GO over-representation analyses, addressing categories from Biological Process, Molecular Function, and Cellular Component. Visualization functions (e.g., dot plots, emap plots) were accessed through enrichplot, with additional customization provided by ggplot2-related libraries (44).

To explore potential epigenetic regulation of transcriptional changes, an integrative analysis was performed by overlaying RNA-seq results with DNA methylation data obtained from the same biological samples. First, differential gene expression analysis was carried out using normalized RNA-seq data. Genes with a fold change >2 or <−2 and adjusted p < 0.05 were classified as significantly differentially expressed genes (DEGs). The list of DEGs was then used to query the DNA methylation dataset generated using the Infinium MethylationEPIC v2.0 BeadChip array. For each DEG, the methylation status of associated CpG sites—particularly those located in gene promoter regions—was extracted. CpG probes corresponding to DEGs were evaluated for differential methylation using the limma package, with significance defined as adjusted p < 0.05 and |Δβ| > 0.2. The analysis aimed to identify genes that were both transcriptionally altered and showed evidence of DNA methylation changes. Particular attention was given to inverse relationships, such as promoter hypermethylation coinciding with downregulation of gene expression, or hypomethylation with upregulation, suggesting potential epigenetic regulation.

The overlap between DEGs and differentially methylated genes (DMGs) was visualized using heatmaps. These integrative results were further subjected to functional enrichment analysis to assess the biological relevance of epigenetically regulated transcriptional changes.

### 2.9 Statistical methods

Statistical analysis was carried out using GraphPad Prism (version 10.6.1. GraphPad Software, USA). For statistical analysis of Western blot and qRT-PCR results, comparisons between two independent cell lines were performed using an unpaired two-tailed Student’s t-test with Welch’s correction to account for unequal variances. Data are presented as mean ± standard deviation (SD), and statistical significance was set at p<0.05. For the analysis of cell viability, a two-way ANOVA with FDR correction followed by Tukey’s *post hoc* test was applied. Adjusted p-values (p.adj<0.05) were considered statistically significant.

## 3 Results

### 3.1 Selecting the dose of vitamins

Assessment of vitamin cytotoxicity was performed using the AlamarBlue assay (Thermo Fisher Scientific). Vitamins were tested at four concentrations (10^-7^ M, 2.5×10^-7^ M, 10^-6^ M, and 2.5×10^-6^ M) and incubated with multiple myeloma cell lines (U266 and MM1S) as well as human fibroblast cells (line NHDF; ATCC, Manassas, VA, USA) for 48 hours under both standard and bone marrow-like conditions. The obtained results were used to determine the appropriate vitamin concentrations for subsequent stages of the experiment. The main objective was to establish a dose that would not be toxic to fibroblast cells. For vitamin D, cytotoxicity toward fibroblasts was observed at a concentration of 2.5×10^-6^ M. Therefore, a lower concentration (10^-6^ M) was selected for further experiments, as it did not exhibit toxic effects on fibroblasts but still reduced cell viability of both multiple myeloma cell lines under both oxygen conditions. The selected concentration of vitamin K was also intended to be non-toxic to fibroblasts; however, no cytotoxicity toward fibroblasts was observed at any of the tested doses. Nevertheless, concentrations of 10^-6^ M and 2.5×10^-6^ M were found to be highly cytotoxic to myeloma cells, resulting in 70–80% cell death. Therefore, the concentration of 2.5×10^-7^ M was chosen, which induced approximately 55% cell death in the MM1S line. A higher level of cell mortality in the tested samples could significantly interfere with data interpretation, leading to misrepresentation of the actual effects of vitamin K. Excessive apoptosis or necrosis may induce secondary alterations in the cellular transcriptome and proteome that are not directly related to the primary mechanism of vitamin K action. Such effects could obscure key signaling or metabolic pathways through which vitamin K exerts its cellular functions, such as the regulation of γ-carboxylation–dependent gene expression or modulation of oxidative stress. Consequently, the observed outcomes might reflect the consequences of cell death rather than the specific biological activity of the vitamin itself.

An additional challenge in experimental design was the selection of appropriate vitamin concentrations under conditions involving two distinct multiple myeloma cell lines cultured both in normoxia and hypoxia. The diverse metabolic backgrounds and differential sensitivity of the cell lines to oxygen availability and nutrient composition suggested that the optimal vitamin concentrations might vary between conditions. However, our aim was to apply uniform concentrations across all experimental variants to enable direct comparison of the results and to evaluate the influence of oxygen conditions on cellular responses to vitamin supplementation. The need to maintain this consistency represented a significant constraint in the experimental design.

### 3.2 Analysis of vitamin D receptor expression in U266 and MM1S cells

This chapter presents the expression level of the vitamin D receptor (VDR) in the cell lines used in the experiment (U266 and MM1S). During bioinformatic analysis of mRNAseq and methylation arrays, substantial differences in the numbers of DEGs and DMPs identified between the cell lines were observed, as described in the respective sections of the Results. Therefore, the following steps were taken to clarify these differences: automated western blot analysis to visualize the semi-quantitative expression of the vitamin D receptor (*VDR*) and qRT-PCR to assess *VDR* gene expression were performed. The results clearly indicate differences in VDR levels between cell lines. Significantly less of this protein was observed in the U266 line, which would correlate with significantly less data obtained from large-scale analyses. These changes may represent an individual characteristic, but paradoxically, they allow for a broader perspective on the effects of vitamins, and vitamin D in particular. The results of the western blot and qRT-PCR analysis are shown in **Figure 1**.

**Figure 1:**
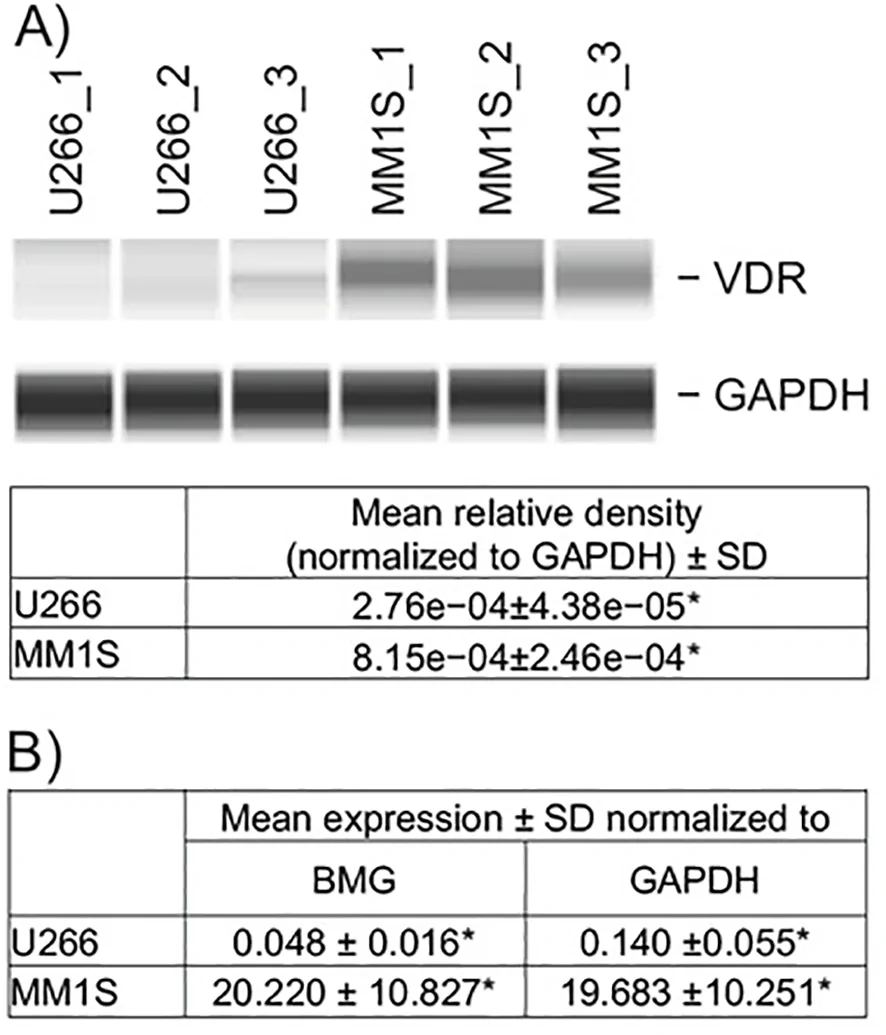
Analysis of vitamin D receptor (*VDR*) expression in multiple myeloma cell lines (U266, MM1S) using: **(A)** Western blot, **(B)** qRT-PCR in three biological replicates. In qRT-PCR analysis, two housekeeping genes (*GAPDH* and *BMG*) were used, and gene expression levels were determined using the ΔΔCt method. *p-value<0.05.

In connection with the results obtained regarding the VDR receptor, the results below focus mainly on comparable data obtained in both lines but also highlight the influence of significant results that were obtained in only one of the lines.

### 3.3 Comparison of the influence of atmospheric and hypoxic conditions on the obtained results of the analyses

#### 3.3.1 Changes in cell viability

The analysis of cell viability showed noticeable differences between both myeloma cell lines depending on oxygen availability and the applied compounds.

For U266 cell line, under standard conditions, two-way ANOVA revealed a significant effect of time (F (3,32)=983.2, p<0.0001) and a significant overall effect of treatment (F (3,32)=2.907, p=0.0497), whereas the interaction between time and treatment was not significant (F (9,32)=1.738, p=0.1208). Under standard conditions, a gradual increase in cell viability was observed over time in all groups of the U266 line. The control group showed the highest cell viability up to day 6. On the 8th day of culture, cell viability in the treatment groups approached the level observed in the control group; however, the differences were not statistically significant.

In bone marrow-like conditions, U266 cells demonstrated a gradual increase in cell viability over time, and overall cell viability values were significantly different from those observed under standard conditions. Two-way ANOVA revealed significant effects of time (F (3,32)=1317, p<0.0001), treatment (F (3,32)=27.68, p<0.0001), and a significant interaction between time and treatment (F (9,32)=6.853, p<0.0001). *Post hoc* Tukey analysis indicated that at early time points (days 3 and 6), the cell viability rate of K2MK-7-treated cells was slightly higher than the control, but these differences were not statistically significant. On day 8, the control group showed the highest cell viability, whereas treatment with K2MK-7 (p.adj=0.007); 25(OH)D (p.adj<0.0001) or in combination with 25(OH)D and K2MK-7 (p.adj<0.0001) resulted in a statistically significant decrease in cell viability compared with the control. The reductions in the Alamar Blue signal were 12%, 20%, and 27%, respectively.

For MM1S cell line, under standard conditions, two-way ANOVA revealed a significant effect of time (F (3,31)=597.4, p<0.0001), a significant overall effect of treatment (F (3,31)=95.99, p<0.0001), as well as the interaction between time and treatment was significant (F (9,31)=13.43, p<0.0001). However, in the MM1S line, the cell viability pattern differed from that observed in U266 cells. Under standard conditions, MM1S cells showed a gradual increase in cell viability over time, reaching the highest values on day 8 (*post hoc* Tukey p.adj<0.0001). Throughout the experiment, the control group maintained the highest cell viability level, while on day 8 of cell culture, treatment with 25(OH)D (p.adj<0.0001), K2MK-7 (p.adj<0.0001), or their combination (p.adj<0.0001) led to a reduction in cell viability (sequentially by 35% and 28%), with the most pronounced decrease observed after combined treatment (44%). Under bone marrow-like conditions, MM1S cells also demonstrated a time-dependent increase in cell viability. Two-way ANOVA revealed significant effects of time (F (3,29)=110.8, p<0.0001), treatment (F (3,29)=13.52, p<0.0001), and a significant interaction between time and treatment (F (9,29)=3.966, p=0.0022). On days 3 and 6, cell viability in the treated groups was similar or slightly lower than that in, while on day 8 the control group again showed the highest cell viability level. Treatment with 25(OH)D (p.adj=0.0016), K2MK-7 (p.adj=0.044), and particularly their combination (p.adj<0.0001) resulted in reductions in the Alamar Blue signal of 34%, 25%, and 51%, respectively. The inhibitory effect was more pronounced than under standard conditions for the last combined treatment group.

In summary, the obtained results differ significantly between pressure and oxygen concentration conditions, with the observed trend indicating stronger effects of the same vitamin doses under bone marrow-like conditions. This is particularly noticeable when analyzing the U266 cell line, where no significant differences were observed after 8 days of cell culture under standard conditions compared to bone marrow-like conditions, where cell viability decreased by 27% after the combined use of both vitamins. Detailed cell viability rate data for each time interval and both cell lines are provided in the **Supplementary Materials** (**Figure 4S**).

Additionally, two-way ANOVA was performed to assess the effects of oxygen pressure conditions (hypoxia vs. atmospheric) and the applied treatments on the analyzed parameter and to determine whether the observed differences were statistically significant. **Figure 2** presents the results, providing a detailed view of the cell viability levels of both cell lines (a) U266 and (b) MM1S on day 8 under the different oxygen conditions and treatments.

**Figure 2:**
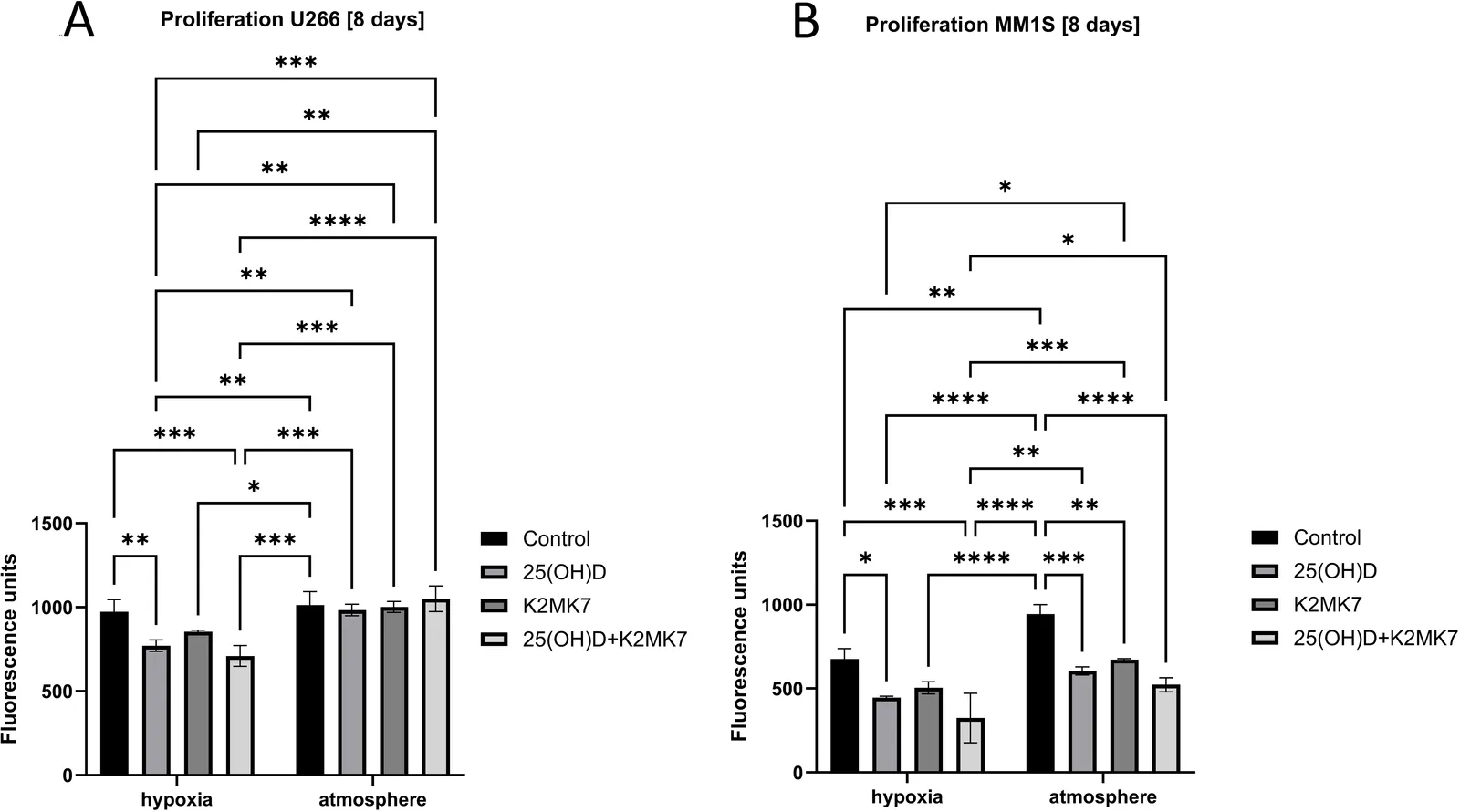
Cell viability analysis using the Alamar Blue assay for a) U266 and b) MM1S myeloma cell lines after 8 days of culture under two oxygen conditions (hypoxia and atmospheric) and vitamin treatment. Statistical analysis was performed using two-way ANOVA followed by Tukey’s *post hoc* test in Graph Pad Prism. *p.adj<0.05; **p.adj<0.01; ***p.adj.<0.001; ****padj<0.0001.

For U266 cell line, statistical analysis revealed a significant effect of oxygen conditions and the applied treatments on the analyzed parameter. A significant interaction between oxygen conditions and treatment was observed (F (3,16)=7.698, p=0.0021), indicating that the effect of the tested compounds depended on oxygen availability. Moreover, a significant main effect of oxygen conditions (F (1,16) = 66.97, p<0.0001) as well as a significant main effect of treatment (F (3,16)=5.756, p=0.0072) were detected. The mean value of the analyzed parameter was significantly lower under bone marrow-like conditions (827.1) compared to standard conditions (1012), with a mean difference of −185.3 (95% CI: −233.4 to −137.3). To identify specific differences between groups, Tukey’s *post hoc* multiple comparisons test was performed. Comparisons of the same treatments between different oxygen conditions revealed significant differences for 25(OH)D (p.adj=0.0047 hypoxia vs. atmosphere) and 25(OH)D+K2MK-7 (p.adj<0.0001 hypoxia vs. atmosphere), indicating a stronger effect of these compounds under bone marrow-like conditions, indicating that oxygen conditions strongly influenced the response, with the combined treatment 25(OH)D+K2MK-7 reducing cell viability by ~27% more relative to the control under hypoxia than under atmospheric oxygen. In the case of K2MK-7, the difference between bone marrow-like and standard conditions did not reach statistical significance (p.adj=0.0728).

Statistical analysis of the MM1S cell line revealed a significant effect of oxygen conditions and applied treatments on the analyzed parameter. Two-way ANOVA showed no significant interaction between oxygen conditions and treatment (F (3,15)=0.7551, p=0.5364), indicating that the effect of the compounds did not depend on oxygen availability. However, significant main effects were observed for both oxygen conditions (F (1,15)=53.80, p<0.0001) and treatment (F (3,15)=33.63, p<0.0001). The mean predicted value under hypoxia was 487.5, compared to 685.6 under standard conditions, with a mean difference of −198.1 (95% CI: −255.7 to −140.6), indicating a significant reduction in cell viability under hypoxia. *Post hoc* Tukey’s multiple comparisons between the same treatments across different oxygen conditions showed significant differences for several groups, including control (p.adj=0.0068 hypoxia vs. atmosphere) and 25(OH)D+K2MK-7 (p.adj=0.0296 hypoxia vs. atmosphere), indicating that oxygen conditions strongly influenced the response, with the combined treatment 25(OH)D+K2MK-7 reducing cell viability by 7.3% more relative to the control under hypoxia than under atmospheric oxygen.

#### 3.3.2 DNA methylation changes

Comprehensive bioinformatics analysis demonstrated that oxygen availability significantly affects DNA methylation profiles in myeloma cell lines (U266 and MM1S). Differential methylation level was determined using the following thresholds: p.adj<0.05, with a beta delta value>0.2, where 1 means full methylation of the gene and 0 means no methylation of the gene. Preliminary analyses comparing control cells cultured under bone marrow-likeand standard conditions revealed notable differences in DNA methylation. In the untreated MM1S cell line under bone marrow-like conditions vs. standard conditions, 103 genes were hypomethylated and 20 genes hypermethylated. In contrast, the U266 cell line showed fewer methylation changes, with only 1 gene hypomethylated and 45 genes hypermethylated. In MM1S cells exposed to hypoxia and treated with vitamins D and K, 4 hypomethylated and 22 hypermethylated genes were identified compared to control cells. These changes occurred predominantly within promoter regions (TSS200 and TSS1500). By contrast, under normoxic conditions, vitamin treatment resulted in markedly different outcomes: 8404 hypermethylated and 306 hypomethylated sites relative to control. In this setting, hypermethylation affected more than 17% of TSS200 and TSS1500 regions, while hypomethylation accounted for nearly 30%. A similar trend was observed in the U266 cell line. Under hypoxia, treatment with vitamins D and K resulted in lack of hypomethylation changes and 83 hypermethylated genes compared to control. These alterations were also enriched in promoter regions, with more than 12% of hypermethylated sites mapping to TSS200 and TSS1500. In contrast, analysis under normoxic conditions revealed 5151 hypermethylated and 118 hypomethylated sites following vitamin treatment relative to control. Among these, hypermethylation was observed in nearly 15% of TSS200 and TSS1500 regions, while hypomethylation was detected in over 30%. Obtaining such extreme results led to the identification of meaningful differences depending on oxygen content and pressure, which significantly influenced the obtained functional results. Heatmaps present genes with the highest variability identified in cells cultured under hypoxia, along with their corresponding beta values obtained for the same genes under normoxic conditions (p.adj<0.05) (**Supplementary Figure 1**). Following vitamin D incubation, only a few dozen genes exhibited methylation changes under hypoxia, whereas several thousand genes showed significant alterations under normoxia. Consequently, the majority of genes altered under normoxic conditions do not display any changes under hypoxia. Therefore, generating heatmaps based on the highest-variability genes identified in normoxia would not be meaningful, as these genes do not undergo significant changes in hypoxia. Moreover, for the small subset of genes that did exhibit changes in hypoxia, the beta values under normoxia differ by at least 20%. This indicates that even the few genes altered in hypoxia have substantially different methylation profiles compared to normoxia, which may significantly impact data interpretation and downstream functional analyses. In summary, bone marrow-like conditions result in a markedly reduced number of methylation changes compared to normoxia, and the differences in beta values for the altered genes highlight the importance of considering oxygen conditions when interpreting results and conducting functional analyses.

#### 3.3.3 Global transcriptome analysis

Bioinformatics data analysis revealed significant differences in gene expression depending on the oxygen conditions used for culturing myeloma cell lines (U266 and MM1S). For all subsequent analyses, the following criteria were applied to qualify gene expression changes: p.adj<0.05; fold change*>*2 for upregulated genes; and fold change<-2 for downregulated genes. Initial comparisons between control cells cultured under bone marrow-like and standard conditions revealed marked transcriptional differences. In the MM1S cell line, 235 genes were downregulated and 99 were upregulated. In contrast, the U266 cell line exhibited fewer differentially expressed genes, with 135 upregulated and only 10 downregulated.

Notably, the changes induced by vitamin treatment varied significantly depending on oxygen concentration and pressure. When comparing MM1S cells treated with vitamins D and K under bone marrow-like conditions vs. control, 2177 genes were found to be upregulated and 2264 downregulated. In the U266 line, only 4 genes were down- and 12 upregulated. Contrary results, although the differences were not as large as those observed for DNA methylation, were observed after incubation of myeloma cells with vitamins under standard conditions. The MMS1 line showed 2422 downregulated and 2153 upregulated genes, while the U266 line showed 253 downregulated and 191 upregulated genes. In the case of the MM1S cell line, the largest differences in gene expression levels comparing hypoxia vs. normoxia were observed for the *PFKFB4, CA9, MIR210HG*, and *TNS1* genes (padj <0.05, fold change log2FC>2). For comparison, in the case of the U266 line, the highest expression variability was identified for the *HK2* (log2FC=0.73, padj=0.0020) and *DDIT3* (log2FC=−0.81; padj=0.0047) genes. Table (**Supplementary Figure 2**) with selected genes with the highest variability and heatmaps for the most important processes (A) cell development; B) cellular response to endogenous stimulus; C) regulation of apoptotic process; D) regulation of cell population proliferation) (**Supplementary Figure 3**) are presented in the supplementary.

Overall, the results indicate significant differences depending on oxygen levels and pressure. Therefore, to optimize experimental procedures and obtain more reliable data, it is important to consider conducting studies under conditions as close to physiological as possible. This is particularly important for cancer cells, as their *in vivo* development environment differs significantly from standard *in vitro* culture conditions.

### 3.4 Functional analysis of the effect of vitamins on DNA methylation and global gene expression

In this study, our main objective was to determine the effects of vitamins D and K under conditions that reflect the *in vivo* microenvironment of multiple myeloma. Therefore, detailed analyses of gene expression, DNA methylation, and functional enrichment were performed and described for physiological oxygen conditions (hypoxia). Results obtained under normoxia were used as a reference for data interpretation and to highlight the importance of employing physiologically conditions in experimental settings. For clarity, part of the functional analyses from normoxia are presented in the Supplementary Information (**Supplementary Figure 1**, **S2**, **S3**). This approach allows the narrative to focus on the most biologically relevant findings while ensuring the completeness of the analysis.

#### 3.4.1 DNA methylation changes

Analysis of global DNA methylation profiles revealed differences between the examined cell lines, although shared features could also be identified. In the interpretation of the data, we focused primarily on genes whose direction of expression changes relative to the control was consistent in both lines, suggesting potentially universal regulatory mechanisms. It should be noted, however, that cell lines may differ at the genetic, cytogenetic, and epigenetic levels, making the identification of highly variable genes observed exclusively in a single line also an important aspect of the analysis. The **Table 1** presents the methylation profiles of selected genes, highlighting similar patterns between the cell lines for genes whose functions may be associated with cancer development.

**Table 1:** Methylation changes in selected genes under bone marrow-like conditions after vitamins incubation in U266 and MM1S cell lines.

Gene	Function/pathway regulated	Expression level and clinical impact (↑/↓, P: progression/R: regression)	MM1S_DK_H vs. MM1S_Control_H	padj.	U266_DK_H vs. U266_Control_H	padj.	Ref.
*PFKP*	Glycolytic enzyme (phosphofructokinase-1), key for cellular metabolism and cell cycle	P ↑ breast, lung, colorectal cancer, R ↑ renal cancer	-0.304	0.018	-0.249	0.004	(45–48)
*BMP4*	TGF-β cytokine, involved in differentiation and EMT	P ↑ melanoma, R ↑ gliomas	-0.233	0.004	-0.115	0.043	(49, 50)
*FOXN3*	Transcription factor, inhibits cell proliferation (tumor suppressor)	P ↓ acute myeloid leukemia, R ↑ hepatocellular carcinoma, P ↑ breast cancer	-0.214	0.016	-0.118	0.007	(51–53)
*ARHGEF10*	Cytoskeleton and cell migration regulator (RhoGEF) (tumor suppressor)	P ↓ pancreatic ductal adenocarcinoma	-0.219	0.003	-0.285	0.005	(54)
*FOXO1*	Transcriptional tumor suppressor, controls apoptosis and metabolism (tumor suppressor)	**R ↑ multiple myeloma,** breast, liver cancer	-0.118	0.074	-0.234	0.043	(55, 56)
*ATM*	DNA repair kinase, cell cycle control (tumor suppressor)	**P ↓ multiple myeloma**, colorectal cancer	-0.117	0.225	-0.299	0.003	(57, 58)
*SERPINB5*	Protease inhibitor (maspin)	P ↑ colorectal cancer, lung adenocarcinoma, P ↓ breast cancer	0.163	0.001	0.350	0.002	(59–61)
*ALK*	Receptor tyrosine kinase (oncogene)	P ↑ lymphoma, myeloma, colorectal, ovarian, pancreatic, renal, and thyroid cancer	0.236	0.002	0.309	0.005	(62)
*ID4*	Regulates differentiation and proliferation, tumor oncogene	P ↑ breast, colorectal cancer,	0.280	0.015	0.172	0.025	(63, 64)
*BCL2L2*	Anti-apoptotic protein, promotes cell survival (oncogene)	P ↑ non-small cell lung cancer, renal cancer	0.279	0.001	0.059	0.003	(65, 66)
*TOX*	Regulator of T cell differentiation (oncogene)	**P ↑ multiple myeloma,** gliomas lymphomas	0.180	0.005	0.283	0.003	(67, 68)
*LPAR3*	LPA receptor, signaling proliferation and migration (oncogene)	**P ↑ multiple myeloma,** R ↑ lung cancer	0.263	0.010	0.242	0.009	(69–71)
*MECOM*	Transcriptional regulator, oncogene (EVI1)	P ↑ ovarian, uterine endometrial cancer, myeloid neoplasms	0.277	0.001	0.276	0.007	(72–74)
*BCL11A*	Regulates hematopoiesis and B cell development (oncogene)	P ↑ breast cancer, acute myeloid leukemia. glioblastoma, neuroblastoma	0.254	0.001	0.283	0.043	(75–77)

A minus sign next to the beta value indicates hypomethylation of the gene relative to the control. The table indicates the expression level observed in individual tumors marked up:↑, down:↓, and information on whether this level is associated with disease progression or regression, i.e. low grade of malignancy and/or lower mortality and/or longer survival time.

Bold values indicate findings related to multiple myeloma. Other disease associations are shown to highlight the potential relevance of these genes to MM.

#### 3.4.2 Global transcriptome analysis

The analysis allowed the distinction of genes with altered expression, that have been previously reported either in MM or in other types of cancer. Genes included in **Table 2** were selected according to the most pronounced differences in expression compared with control cells and their known relevance for the biology of myeloma or other tumor cells. Additionally, **Supplementary Figure 5S** presents the expression levels of these genes in untreated and vitamin-treated cells, together with the corresponding fold changes, allowing their regulation to be evaluated in relation to the basal expression levels in each cell line. The effect of vitamins on gene expression was analyzed in a manner similar to the methylation array. The focus was primarily on results consistent across both cell lines, but significant expression changes observed exclusively in the MM1S line (**Table 3**) were also noted. Two genes, *BBC3* and *KLF6*, are particularly noteworthy, as their expression increased in both lines following vitamin treatment. These genes are known MM suppressors, and their upregulation may have a beneficial impact on treatment outcomes and inhibit disease progression. However, the most pronounced changes were observed in the MM1S line alone. The variability in gene expression after vitamin treatment compared to control was remarkable, with fold changes of 1089.51 for *ABCA1*, 95.76 for *ADAMTS9*, and 59.34 for *RUNX1T1*. To facilitate interpretation of these large fold-change values, mean rlog-transformed expression values for the control and combined vitamin D3 and vitamin K2 treatment groups are provided in **Table 3**. Particularly high fold changes may occur when baseline expression in control cells is low. Of particular note is the *ABCA1* gene, which encodes a protein transporter primarily involved in the removal of cholesterol and lipids from cells. Increased *ABCA1* gene expression may potentially limit tumor development by inducing apoptosis and limiting cell proliferation associated with lipid metabolism. This opens up a new direction for research on MM progression. The remaining genes included in the table also exhibited substantial variability, with fold changes greater than 5. All reported results were statistically significant (padj < 0.05). Some of the genes highlighted, including *ADAMTS9, CD79A, HGF, LTBR*, and *GPRC5D*, are already referenced in the literature in relation to MM. However, the majority of these genes are primarily known for their roles in regulating the progression of other cancers, suggesting significant potential for further investigation in the context of MM.

**Table 2:** Expression of selected genes under bone marrow-like conditions after vitamins incubation in U266 and MM1S cell lines.

Gene	Function/pathway regulated	Expression level and clinical impact (↑/↓, P: progression/R: regression)	MM1S_DK_H vs. MM1S_Control_H	padj.	U266_DK_H vs. U266_Control_H	padj.	Ref.
*PER1*	Circadian rhythm regulation, DNA damage response/p53 pathway	P ↓ colon cancer R ↑ AML, oral squamous cell carcinoma, ↓ R breast cancer	2.41	6.72E-52	2.18	1.59E-02	(78–80)
*BBC3*	Pro-apoptotic protein, p53 target/Apoptosis, p53	P ↑ prostate cancer **R ↑ multiple myeloma**	2.23	9.69E-20	2.04	1.47E-04	(81, 82)
*DAPK1*	Serine/threonine kinase, apoptosis/p53, autophagy	P ↑ Gastric cancer, breast cancer P ↓ renal cell cancer	0.83	1.56E-03	1.69	1.73E-02	(83–85)
*MXD1*	Transcriptional repressor, Myc antagonist/Myc pathway	P ↓ esophageal squamous cell carcinoma	2.21	1.70E-28	1.67	9.29E-03	(86)
*KLF6*	Tumor suppressor transcription factor/TGF-β, cell cycle	**P ↓ multiple myeloma**, P ↑ protate cancer,	1.33	2.64E-09	1.55	3.02E-02	(87, 88)
*FDFT1*	Cholesterol biosynthesis/Mevalonate pathway	P ↑ hepatocellular carcinoma, tongue squamous cell carcinoma, R ↑ renal cell carcinoma	-2.08	4.70E-40	-1.47	3.02E-02	(89–91)
*IDH1*	Metabolism, epigenetic regulation/TCA cycle, HIF1α	R ↓ glioblastoma	-2.95	1.65E-50	-1.64	1.00E-02	(92)
*MSMO1*	Cholesterol biosynthesis/Mevalonate pathway	P ↑ esophageal cancer, cervical cancer	-2.95	2.84E-86	-1.70	1.20E-06	(93, 94)

Fold-change values are presented as linear fold changes. A minus sign next to the fold value indicates a decrease in gene relative to the control. Additionally, the table indicates the expression level observed in individual tumors marked up:↑, down:↓, and information on whether this level is associated with disease progression or regression, i.e. low grade of malignancy and/or lower mortality and/or longer survival time.

Bold values indicate findings related to multiple myeloma. Other disease associations are shown to highlight the potential relevance of these genes to MM.

**Table 3:** Expression of selected genes under bone marrow-like conditions after vitamins incubation in MM1S cell line.

Gene	Function/pathway regulated	Expression level and clinical impact (↑/↓, P: progression/R: regression)	Mean normalized expression – Control	Mean normalized expression – VD+VK	Fold Change	padj.	Ref.
*ABCA1*	Cholesterol transport. regulates proliferation/Cholesterol efflux. AKT/mTOR (tumor suppressor)	P ↑ triple negativ breast cancer P ↓ chronic myelomonocytic leukemia. melanoma	0.829	904.076	1089.51	6.93E-10	(95–97)
*ADAMTS9*	Tumor suppressor. inhibits migration and angiogenesis/AKT/mTOR. ECM remodeling	**P ↓ multiple myeloma** P ↓ gastric cancer	0.338	32.463	95.76	2.33E-06	(98, 99)
*RUNX1T1*	Suppresses tumor growth by regulating gene expression and cell proliferation pathways/RUNX1 pathway. epigenetic transcriptional regulation	P ↓ pancreatic endocrine tumors. breast cancer. colorectal cancer.	0.677	40.232	59.34	7.55E-07	(100)
*DMBT1*	Tumor suppressor. controls adhesion and migration/Cell adhesion. MAPK regulation	P ↓ prostate cancer. glioblastoma multiforme. lung cancer	21.349	358.148	16.77	6.72E-69	(101, 102)
*ARHGAP24*	Suppresses migration and invasion/Rho GTPases (Rac1. Cdc42)	P ↓ hepatocellular carcinoma. colorectal cancer	11.623	116.610	10.03	4.28E-23	(103, 104)
*DIRC3*	Tumor suppressor lncRNA/Gene expression regulation. epigenetic mechanisms	P ↓ thyroid cancer. melanoma	4.531	39.972	8.82	1.81E-08	(105, 106)
*MTSS1*	Suppresses metastasis. controls cytoskeleton/Rac1/Cdc42. actin remodeling	R ↑ breast cancer P ↓ chronic myeloid leukemia	6.658	43.658	6.55	1.48E-08	(107, 108)
*CD79A*	B-cell marker. regulates BCR signaling/BCR signaling. PI3K/AKT	**P ↓ multiple myeloma**	53.415	337.852	6.32	4.79E-50	(109)
*IGFBP1*	Regulates proliferation and migration/IGF/IGF1R. PI3K/AKT	P↓ hepatocellular carcinoma	3.354	20.691	6.16	2.36E-04	(110)
*CAPN9*	Potential regulator of migration and apoptosis/Calpain-mediated signaling	P ↓ gastric cancer	1.587	9.296	5.85	1.96E-02	(111)
*GADD45A*	DNA damage response. apoptosis control/p38/JNK. p53. DNA damage response	P ↑ breast cancer. P ↓ acute myeloid leukemia	855.516	4803.369	5.61	2.07E-237	(112–114)
*TNFSF15*	Angiogenesis. immune modulation/TNF receptor signaling	R ↓ esophageal cancer P ↑ oral cavity squamous cell carcinoma	52.478	1.672	-31.37	6.60E-13	(115, 116)
*STYK1*	Tyrosine kinase activity/MAPK signaling	P ↑ colorectal cancer. gallbladder carcinoma	5.612	0.287	-19.52	1.24E-02	(117–119)
*HGF*	Hepatocyte growth factor. proliferation/c-MET receptor signaling	**P ↑ multiple myeloma.** hepatocellular carcinoma	14.551	0.793	-18.33	1.74E-04	(120, 121)
*IFIT1*	Interferon-induced protein. antiviral response/JAK-STAT signaling	P ↑ oral squamous cell carcinoma. AML	96.785	5.359	-18.05	5.03E-25	(122, 123)
*RAB27B*	Vesicle trafficking. exocytosis/Cell migration	P ↑ breast cancer. ovarian cancer	29.0471	1.695	-17.13	3.76E-08	(124, 125)
*MX2*	Interferon-induced GTPase/Immune response	P ↑ renal cell carcinoma	129.499	9.661	-13.4	2.46E-32	(126)
*LTBR*	Lymphotoxin beta receptor. immune response/NF-κB signaling	**P ↑ multiple myeloma.** liver cancer	6.442154716	0.560374219	-11.49	1.86E-02	(127–129)
*GPRC5D*	G protein-coupled receptor. cell signaling/Signal transduction	**P ↑ multiple myeloma**	4150.794	562.337	-7.38	5.67E-220	(130)
*EPHA1*	Receptor tyrosine kinase/Cell migration	P ↑ renal cell carcinoma. colorectal cancer	6.514	1.175	-5.54	4.46E-02	(131, 132)
*DDR1*	Collagen receptor. ECM signaling/Cell adhesion & migration	P ↑ head and neck cancer. liver cancer.	16.702	3.086	-5.41	6.18E-04	(133)
*EPHA6*	Receptor tyrosine kinase/Cell migration	P ↑ breast cancer P ↓ glioma	8.931	1.704	-5.24	1.72E-02	(134, 135)
*SPARC*	ECM protein. cell adhesion/ECM remodeling & angiogenesis	P ↑ bladder cancer	1084.523	209.658	-5.17	4.89E-123	(136)

Fold-change values are presented as linear fold changes. A minus sign next to the fold value indicates a decrease in gene relative to the control. Additionally, the table indicates the expression level observed in individual tumors marked up:↑, down:↓, and information on whether this level is associated with disease progression or regression, i.e. low grade of malignancy and/or lower mortality and/or longer survival time. Bold values indicate findings related to multiple myeloma. Other disease associations are shown to highlight the potential relevance of these genes to MM.

GSEA analysis (**Figure 3**) revealed that, in both cell lines, treatment with vitamins led to a reduction in processes mainly related to the cell cycle, such as nuclear division, chromatid separation, establishment of chromosome localization, chromosome separation, and segregation. Interestingly, additional analysis using pathfindR (padj<0.05, fold>2) (**Table 4**) showed that in both cell lines the cell cycle pathway was markedly overrepresented (which in pathfindR analysis indicates that many genes are involved in a given process, without specifying the direction of regulation), along with other pathways regulating the cell cycle, such as p53, MAPK, and FoxO, confirming the results obtained from GSEA. The steroid biosynthesis pathway was the most prominent, achieving the highest fold enrichment in the study (U266:148.83; MM1S:5.00). This indicates that genes associated with this pathway are highly enriched in the list of statistically relevant genes, highlighting its key role in regulating biological pathways in the analyzed cell lines. Vitamin D and K may have a substantial impact on steroid metabolism and cellular functions, including proliferation, differentiation, and survival. Another important finding was the overrepresentation of the IL-17 signaling pathway, which, although showing a high fold enrichment (16.83), was observed only in the U266 cell line.

**Figure 3:**
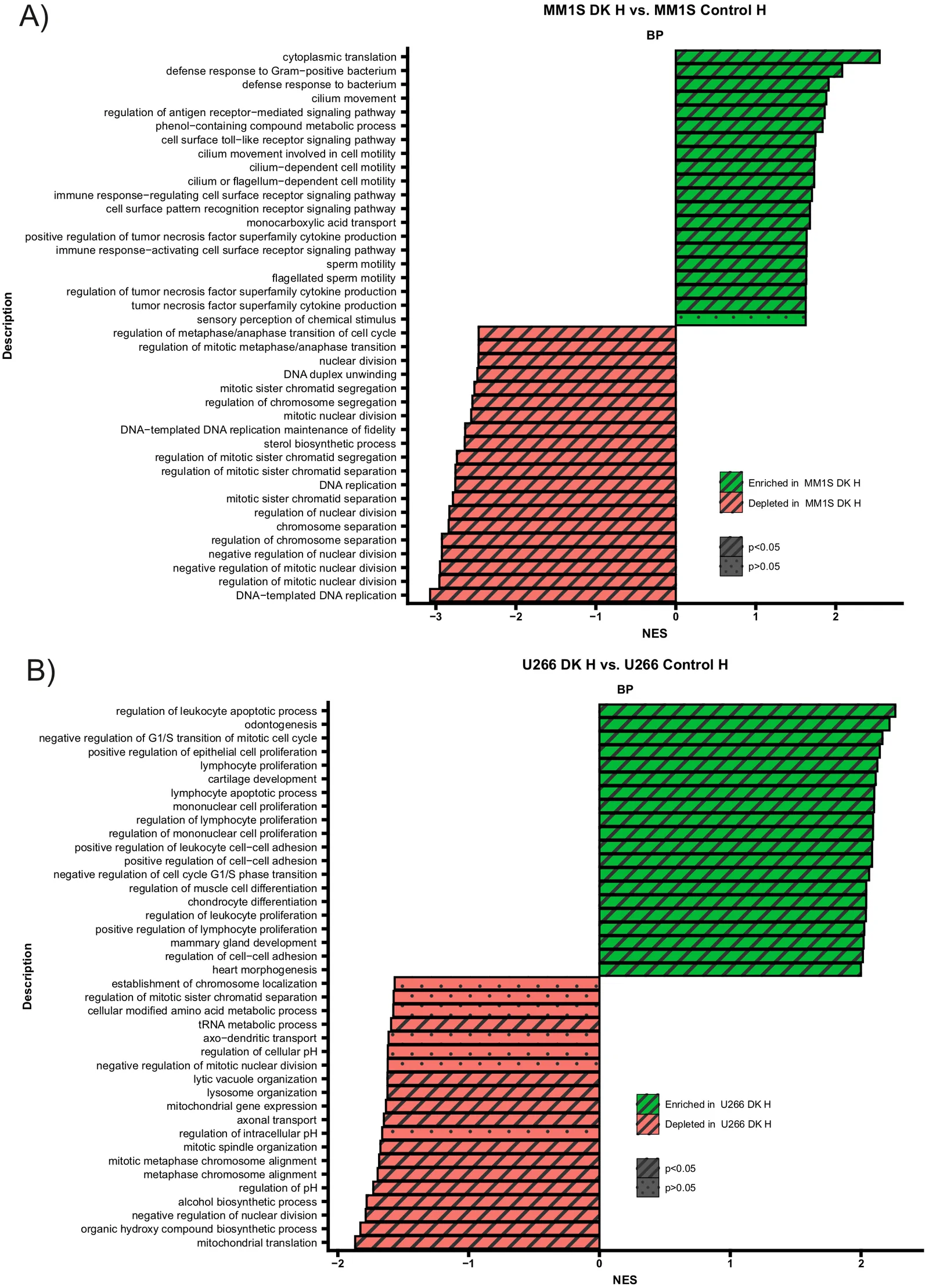
GSEA analysis in **(A)** MM1S and **(B)** U266 cell lines after vitamin treatment. The X-axis shows NES (Normalized Enrichment Score) values, reflecting the strength and direction of pathway enrichment: NES > 0 indicates enriched processes, NES < 0 indicates depleted processes. The Y-axis shows biological processes. Point colors represent the direction of change (green – enriched, red – depleted), and appropriate patterns indicate statistical significance.

**Table 4:** Pathway enrichment analysis using pathfindR (padj<0.05, fold>2) in U266 and MM1S cell lines after vitamin treatment.

Biological process/pathway	U266 [fold enrichement]	MM1S [fold enrichement]
p53 signaling pathway	40.40	2.91
Apoptosis	23.18	2.06
MAPK signaling pathway	10.71	1.52
Chronic myeloid leukemia	19.64	1.70
IL-17 signaling pathway	16.83	–
GnRH signaling pathway	16.83	1.86
Central carbon metabolism in cancer	22.09	1.59
Growth hormone synthesis, secretion and action	13.21	1.90
Neurotrophin signaling pathway	12.85	1.60
FoxO signaling pathway	11.68	1.68
Osteoclast differentiation	11.40	1.64
Ubiquitin mediated proteolysis	22.44	1.02
C-type lectin receptor signaling pathway	15.20	1.46
Cell cycle	9.75	2.48
Cellular senescence	9.62	2.17
Viral carcinogenesis	9.49	1.73
Transcriptional misregulation in cancer	9.18	1.67
Steroid biosynthesis	148.83	5.00
DNA replication	–	4.59

The table shows fold enrichment values, indicating the degree of overrepresentation of each pathway in the analyzed gene list (without specifying the direction of up- or down-regulation), along with the names of selected pathways.

### 3.5 Integrative analysis

An integrative analysis comparing methylation and transcription profiles revealed only limited changes following incubation with vitamins under bone marrow-like conditions. The objective of the analysis was to identify statistically significant alterations, based on the assumption that hypermethylation suppresses gene expression, whereas hypomethylation enhances it (**Figure 4**). The analysis criteria were as follows: fold change expression>2; beta value>0.2, padj<0.05. Within this framework, only two genes, *MCOLN3* and *CLSTN2*, exhibited a concurrent increase in methylation and decreased their expression. Notably, hypermethylation of the *MCOLN3* gene was detected in the promoter region (TSS1500), which increases the likelihood of a direct impact of this modification on its expression levels. The *MCOLN3* gene encodes the protein mucolipin-3, which functions as an ion channel primarily located in lysosomes and endosomes. It is involved in endosomal transport and autophagy, facilitating the fusion of endosomes with lysosomes and the degradation of cellular components. Dysregulation of its function may lead to disturbances in calcium signaling and degradative processes, thereby promoting tumorigenic transformation. Through the modulation of autophagy and interaction with key pathways such as mTOR, *MCOLN3* may support the adaptation of cancer cells to conditions of metabolic stress and hypoxia, enhancing their survival and tumor progression. Increasing evidence also suggests that *MCOLN3* may play an important role in the pathogenesis of multiple myeloma. Studies have shown that its expression is significantly elevated in patients with both newly diagnosed and relapsed or refractory myeloma, compared with healthy individuals and those in remission. In contrast, the integrative analysis performed after incubation of myeloma cells with vitamins under normoxic conditions revealed a markedly higher number of correlations using the same analytical criteria. Specifically, for the MM1S cell line, 312 associations were identified, of which 148 were located in promoter regions (TSS200 and TSS1500) as well as in the 5′UTR. For the U266 cell line, 26 correlations were identified, including 10 in promoter regions and the 5′UTR.

**Figure 4:**
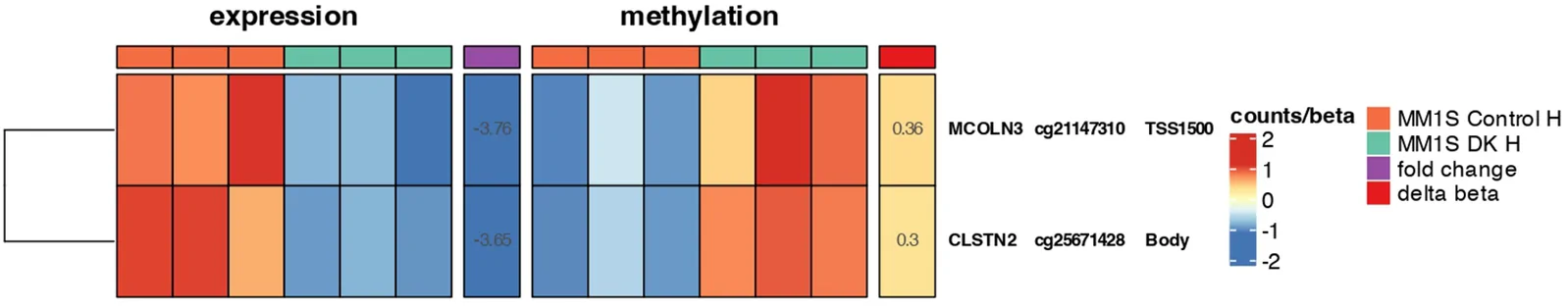
Heatmap showing the integration analysis of results obtained under bone marrow-like conditions for the MM1S line for mRNAseq (fold change) and DNA methylation array (beta value) (padj<0.05). Negative fold-change values indicate a decrease in gene expression; the negative sign denotes the direction of change.

Our study revealed clear differences between standard and bone marrow-like conditions at multiple levels, including DNA methylation, gene expression, enrichment analyses, and integrative comparisons, even though the vitamin dose and incubation time were the same. Under normoxia, more correlations and changes in promoter regions were observed, while hypoxia resulted in fewer detectable alterations. These results suggest that oxygen levels strongly influence cellular responses, affecting transcriptional and epigenetic regulation as well as pathway activity. This emphasizes the importance of maintaining culture conditions that closely resemble the physiological environment to ensure that *in vitro* findings are reliable and reflective of *in vivo* biology.

## 4 Discussion

Multiple myeloma still remains incurable yet due to modern treatment strategies the prognosis and clinical outcomes have significantly improved. Nonetheless, despite the recent progress, there is room for improvement and further preclinical and clinical research. The aim of this study was to investigate the effect of VD and VK upon MM cells under standard and bone marrow-like conditions to unveil molecular and epigenetic aspects associated with MM cells cell viability and survival.

Bone marrow microenvironment is crucial for understanding the biology of MM, mechanisms of myelomagenesis as well as disease progression, relapse and development resistance to anti-MM treatment (137). Although there were multiple preclinical studies being conducted, many were conducted under atmospheric oxygen pressure and failed to recognize the hypoxic environment which is a hallmark of MM niche (138). In this study, we demonstrated that gene expression, methylation profile, as well as proliferative potential of MM cells differed between standard and bone marrow-like conditions. This finding is crucial for the design of future *in-vitro* MM studies. Having considered that hypoxia better represents MM microenvironment and affects epigenetic alterations, gene expression and proliferative potential, results obtained in standard oxygen conditions may be inaccurate. Therefore, due to the abovementioned rationale, in *in-vitro* MM studies bone marrow-like conditions should be preferred, and perhaps in the future, constitute a new standard in study design and methodology in MM research.

VD is not a classical vitamin. From the standpoint of its molecular mechanism of action, it should be rather considered as a prohormone synthesized in the skin upon exposure to ultraviolet light in addition to dietary intake (139). Activation of VD requires two hydroxylations. The first, in which 25(OH)D3 is synthesized, occurs in the liver. Subsequently, 25(OH)D3 (calcidiol) is subjected to the second hydroxylation in the kidneys, mainly in the proximal tubule. The final product of these reactions is 1,25(OH)2D3 (calcitriol), being the most biologically active form of VD (139). The classical role of VD is the regulation of calcium and phosphate homeostasis, and it is therefore crucial for bone metabolism and health.

The role of VD in calcium metabolism is very well-established. Nonetheless, so-called non-classical actions, i.e., not directly related to the regulation of calcium-phosphate metabolism and bone health, are the subject of intensive ongoing research. In the first place, one should consider that VDR is expressed in most human cells (140). Therefore, it may be assumed that VD influences the metabolism and function of various cell types via regulating gene expression, exhibiting numerous pleiotropic effects. Besides genomic action mediated by VDR, VD also exhibits non-genomic effects, believed to be mediated by membrane receptors (141, 142). Non-genomic actions are predominantly associated with activating various signaling molecules, the subsequent generation of second messengers, and the opening of ion channels (143). To highlight the rationale for investigating VD in MM, it should be mentioned that VDR is also expressed on MM cells. In the present study, the expression of VDR in U266 and MM1S cells was confirmed at both the mRNA and protein levels using qRT-PCR and automated Western blot analysis, respectively (**Figure 1**). Differences in VDR expression levels and the activity of VDR-dependent signaling pathways between MM cell lines may partially contribute to their distinct responses to combined vitamin D3 and vitamin K2 treatment. In addition, the divergent transcriptomic and DNA methylation profiles observed between U266 and MM1S cells may reflect cell-line-specific differences in baseline epigenetic states, chromatin accessibility, and other regulatory mechanisms affecting gene expression.

In the first step of our experiment, U266 and MM1 cell lines were cultured with VD under standard and bone marrow-like conditions. In these two very different environments, it was observed that VD affects the cell viability potential of MM cells. More precisely, VD decreases the cell viability rate of MM cells both in standard and bone marrow-like conditions. Although differences in proliferative potential, methylation profiles, and gene expression patterns were observed between the U266 and MM1S cell lines, these differences may reflect cell-line-specific molecular characteristics, potentially including differences in genetic and epigenetic backgrounds. A deeper analysis of methylation and gene expression profile revealed the alterations in genes associated with cancer development and progression, not only in MM but also in solid tumors. **Table 1** presents the methylation profiles of selected genes, revealing similar patterns between both (U266 and MM1S) investigated cell lines. The identified genes are highly associated with cancer development. Previously documented evidence regarding their involvement in multiple myeloma primarily applies to the *ATM* (57, 58), *FOXO1* (55, 56), *TOX* (67, 68), *LPAR3* (69–71) genes. Nevertheless, the other genes analyzed such as *BCL11A* (75–77), *MECOM* (72–74), *BCL2L2* (65, 66), *ARHGEF10* (54), *FOXN3* (51–53) may also play important roles, although their significance in multiple myeloma has not yet been established. In other malignancies, these genes have been associated with disease progression and metastasis, suggesting their potential functional relevance. Nonetheless, their functional importance in MM requires additional studies. Interestingly, genes commonly recognized as oncogenes (including *TOX* (67, 68), *LPAR3, BCL2L2* (65, 66), *MECOM* (72–74), and *BCL11A* (75–77) were hypermethylated in the aftermath of vitamin treatment.

Another important finding was the overrepresentation of the IL-17 signaling pathway, after vitamin treatment which, although showing a high fold enrichment (16.83), was observed only in the U266 cell line. From the perspective of MM, IL-17 has been reported to act as a potential growth-promoting factor for MM cells. Moreover, this pathway may influence the bone marrow microenvironment, potentially supporting proliferation and survival of malignant cells, contributing to promote disease progression, and modulating the inflammatory response (144). Having considered the anti-MM activity of VD, the overexpression of IL-17 related signaling might represent a potential cellular response to the effects of vitamin treatment. Similar observation was made in MM patients achieving deep and long-lasting response to lenalidomide (145) in whom increased activity of the IL-17 axis was proposed as a potential mechanism that might enhance IL-17-related signaling in MM cells and potentially counteract the anti-MM effects of lenalidomide. Although in the context of this study the role of IL-17 signaling remains unclear, this observation may provide further support for investigating the potential role of IL-17 signaling in MM.

The above observations from this experiment alongside other *in vitro* research (146), including these conducted by our team (9, 10, 20), and human studies (147–150) strongly suggests the rationale for restoring the VD status in MM patients to boost the immune system, enhance the efficacy of anti-MM treatment regimen and improve overall prognosis.

## 5 Conclusions

The conducted studies demonstrated both the effects of vitamins D and K on myeloma cells and the importance of maintaining oxygen conditions during cell culture and throughout the experiment to better reflect their natural microenvironment.

Hypoxia within the tumor microenvironment affects numerous biological processes, including enhancing cellular stress resistance, promoting angiogenesis, shifting metabolism toward anaerobic glycolysis, and influencing gene expression and epigenetic modifications. The results of our *in vitro* analyses suggest that the limited number of changes observed under bone marrow-like conditions may partially reflect adaptive cellular responses to low oxygen levels, including metabolic, transcriptional, and epigenetic changes, which may influence the response of cancer cells to treatment. This emphasizes the importance of conducting experiments under conditions that closely mimic physiological environments to ensure that the results are biologically relevant and more accurately reflect the actual behavior of cancer cells.

Focusing on the results obtained after incubation with vitamins under bone marrow-like conditions, a number of genes, such as *ABCA1, ADAMTS9, RUNX1T1*, showed pronounced expression changes specifically in the MM1S cell line. These findings should therefore be considered cell-line-specific and may represent potential candidates for further investigation rather than generalizable mechanisms of vitamin action in multiple myeloma. Notably, one of the most intriguing findings is the observed association between hypermethylation of the *MCOLN3* gene and a reduction in its expression. This may suggest a potential pathway associated with the antitumor effects of vitamins D and K but may also provide a potential direction for future gene-targeted therapies.

## 6 Study limitations

We acknowledge that this study has certain limitations. An additional limitation of the omics component is that transcriptomic and methylation analyses were performed only for the combined 25(OH)D+K2MK-7 treatment group and the corresponding control group. Although the effects of each vitamin individually were assessed in the cell viability experiments, the individual transcriptomic and epigenetic contributions of 25(OH)D and K2MK-7 could not be distinguished in the omics analyses. The experimental design did not include a wide range of vitamin concentrations or incubation intervals, which could have better revealed the potential effects of the vitamins, particularly under bone marrow-like conditions. Another limitation is that VDR expression was not assessed under bone marrow-like conditions. Therefore, we cannot determine whether the different cellular responses observed under these conditions, particularly the weaker transcriptional response in U266 cells, are associated with changes in VDR expression. Further studies assessing VDR expression under bone marrow-like conditions are warranted to clarify this potential mechanism. Furthermore, the analysis was limited to two cell lines, and expanding the study to include myeloma lines with diverse genetic backgrounds would allow for a more comprehensive understanding of the observed effects. In particular, the markedly reduced VDR expression observed in the U266 cell line represents a cell-line-specific finding that may contribute to the distinct responses observed between MM cell lines. This observation further highlights the genetic and epigenetic heterogeneity among MM cells and reinforces the need to use multiple myeloma cell lines with diverse molecular backgrounds in future *in vitro* studies. As an *in vitro* cell-line-based study, the translational relevance of the findings remains to be validated in primary patient-derived samples and *in vivo* models. Future research should address these aspects to confirm and expand upon our findings. Nonetheless, the main goal of the study—to demonstrate differences arising from varying oxygen concentrations and pressures—was achieved. Significant differences were observed, clearly confirming the importance of replicating a tissue’s natural microenvironment in *in vitro* research.

## Data Availability

The data presented in the study are deposited in the RepOD (Repository of Open Data) repository, accession number DOI: 10.18150/JDYGJR.

## References

[1] Malard F Neri P Bahlis NJ Terpos E Moukalled N Hungria VTM . Multiple myeloma. Nat Rev Dis Primers. (2024) 10:45. doi: 10.1038/s41572-024-00529-7 38937492

[2] van de Donk NWCJ Palumbo A Johnsen HS Engelhardt M Gay F Gregersen H . The clinical relevance and management of monoclonal gammopathy of undetermined significance and related disorders: recommendations from the European Myeloma Network. Haematologica. (2014) 99:984. doi: 10.3324/haematol.2013.100552 24658815 PMC4040895

[3] Swerdlow SH Campo E Pileri SA Harris NL Stein H Siebert R . The 2016 revision of the World Health Organization classification of lymphoid neoplasms. Blood. (2016) 127:2375–90. doi: 10.1182/blood-2016-01-643569 26980727 PMC4874220

[4] Rajkumar SV Dimopoulos MA Palumbo A Blade J Merlini G Mateos MV . International Myeloma Working Group updated criteria for the diagnosis of multiple myeloma. Lancet Oncol. (2014) 15:e538–48. doi: 10.1016/s1470-2045(14)70442-5 25439696

[5] González D van der Burg M García-Sanz R Fenton JA Langerak AW González M . Immunoglobulin gene rearrangements and the pathogenesis of multiple myeloma. Blood. (2007) 110:3112. doi: 10.1182/blood-2007-02-069625 17634408

[6] Chesi M Nardini E Brents LA Schröck E Ried T Kuehl WM . Frequent translocation t(4;14)(p16.3;q32.3) in multiple myeloma is associated with increased expression and activating mutations of fibroblast growth factor receptor 3. Nat Genet. (1997) 16:260. doi: 10.1038/ng0797-260 9207791 PMC3901950

[7] Lauring J Abukhdeir AM Konishi H Garay JP Gustin JP Wang Q . The multiple myeloma associated MMSET gene contributes to cellular adhesion, clonogenic growth, and tumorigenicity. Blood. (2008) 111:856. doi: 10.1182/blood-2007-05-088674 17942756 PMC2200833

[8] Rajkumar SV . Multiple myeloma: 2024 update on diagnosis, risk-stratification, and management. Am J Hematol. (2024) 99:1802–24. doi: 10.1002/ajh.27422 38943315 PMC11404783

[9] Łuczkowska K Kulig P Baumert B Machaliński B . Vitamin D and K supplementation is associated with changes in the methylation profile of U266-multiple myeloma cells, influencing the proliferative potential and resistance to bortezomib. Nutrients. (2023) 16:142. doi: 10.3390/nu16010142 38201971 PMC10780809

[10] Łuczkowska K Kulig P Baumert B Machaliński B . The evidence that 25(OH)D3 and VK2 MK-7 vitamins influence the proliferative potential and gene expression profiles of multiple myeloma cells and the development of resistance to bortezomib. Nutrients. (2022) 14:5190. doi: 10.3390/nu14235190 36501221 PMC9736786

[11] Caprio C Sacco A Giustini V Roccaro AM . Epigenetic aberrations in multiple myeloma. Cancers (Basel). (2020) 12:2996. doi: 10.3390/cancers12102996 33076518 PMC7602661

[12] De Miguel MP Alcaina Y de la Maza DS Lopez-Iglesias P . Cell metabolism under microenvironmental low oxygen tension levels in stemness, proliferation and pluripotency. Curr Mol Med. (2015) 15:343–59. doi: 10.2174/1566524015666150505160406 25941818

[13] Dawes CS Konig H Lin CC . Enzyme-immobilized hydrogels to create hypoxia for *in vitro* cancer cell culture. J Biotechnol. (2017) 248:25–34. doi: 10.1016/j.jbiotec.2017.03.007 28284922

[14] Broxmeyer HE O'Leary HA Huang X Mantel C . The importance of hypoxia and extra physiologic oxygen shock/stress for collection and processing of stem and progenitor cells to understand true physiology/pathology of these cells ex vivo. Curr Opin Hematol. (2015) 22:273–8. doi: 10.1097/MOH.0000000000000144 26049746 PMC4721218

[15] Bowyer C Lewis AL Lloyd AW Phillips GJ Macfarlane WM . Hypoxia as a target for drug combination therapy of liver cancer. Anticancer Drugs. (2017) 28:771–80. doi: 10.1097/CAD.0000000000000516 28542038 PMC5515631

[16] Hicklin DJ Ellis LM . Role of the vascular endothelial growth factor pathway in tumor growth and angiogenesis. J Clin Oncol. (2005) 23:1011–27. doi: 10.1200/JCO.2005.06.081 15585754

[17] Brahimi-Horn MC Chiche J Pouysségur J . Hypoxia and cancer. J Mol Med (Berl). (2007) 85:1301–7. doi: 10.1007/s00109-007-0281-3 18026916

[18] Strese S Fryknäs M Larsson R Gullbo J . Effects of hypoxia on human cancer cell line chemosensitivity. BMC Cancer. (2013) 13:331. doi: 10.1186/1471-2407-13-331 23829203 PMC3707755

[19] Lopez AG Kerlan V Desailloud R . Non-classical effects of vitamin D: Non-bone effects of vitamin D. Ann Endocrinol (Paris). (2021) 82:43–51. doi: 10.1016/j.ando.2020.12.002 33279474

[20] Kulig P Łuczkowska K Bielikowicz A Zdrojewska D Baumert B Machaliński B . Vitamin D as a potential player in immunologic control over multiple myeloma cells: Implications for adjuvant therapies. Nutrients. (2022) 14:1802. doi: 10.3390/nu14091802 35565770 PMC9105067

[21] Jeon SM Shin EA . Exploring vitamin D metabolism and function in cancer. Exp Mol Med. (2018) 50:1–14. doi: 10.1038/s12276-018-0038-9 29657326 PMC5938036

[22] Bikle DD . Extraskeletal actions of vitamin D. Ann N Y Acad Sci. (2016) 1376:29–52. doi: 10.1111/nyas.13219 27649525 PMC5031366

[23] Wang H Chen W Li D Yin X Zhang X Olsen N . Vitamin D and chronic diseases. Aging Dis. (2017) 8:346–53. doi: 10.14336/AD.2016.1021 28580189 PMC5440113

[24] Chauss D Freiwald T McGregor R Yan B Wang L Nova-Lamperti E . Autocrine vitamin D signaling switches off pro-inflammatory programs of TH1 cells. Nat Immunol. (2022) 23:62–74. doi: 10.1038/s41590-021-01080-3 34764490 PMC7612139

[25] Karlic H Varga F . Impact of vitamin D metabolism on clinical epigenetics. Clin Epigenet. (2011) 2:55–61. doi: 10.1007/s13148-011-0021-y 22704269 PMC3365599

[26] Snegarova V Naydenova D . Vitamin D: a review of its effects on epigenetics and gene regulation. Folia Med (Plovdiv). (2020) 62:662–7. doi: 10.3897/folmed.62.e50204 33415918

[27] Wawrzyniak A Mincer-Chojnacka I Kalicki B Lipińska-Opałka A Jobs K Stelmasiak A . Plejotropowe działanie witamin D i K (ang. Pleiotropic effects of vitamins D and K). Pediatr Med Rodz. (2015) 11:374–81. doi: 10.15557/PiMR.2015.0035

[28] Duan F Mei C Yang L Zheng J Lu H Xia Y . Vitamin K2 promotes PI3K/AKT/HIF-1α-mediated glycolysis that leads to AMPK-dependent autophagic cell death in bladder cancer cells. Sci Rep. (2020) 10:7714. doi: 10.1038/s41598-020-64880-x 32382009 PMC7206016

[29] Xu W Wu H Chen S Wang X Tanaka S Sugiyama K . Cytotoxic effects of vitamins K1, K2, and K3 against human T lymphoblastoid leukemia cells through apoptosis induction and cell cycle arrest. Chem Biol Drug Des. (2020) 96:1134–47. doi: 10.1111/cbdd.13696 32305047

[30] Yokoyama T Miyazawa K Naito M Toyotake J Tauchi T Itoh M . Vitamin K2 induces autophagy and apoptosis simultaneously in leukemia cells. Autophagy. (2008) 4:629–40. doi: 10.4161/auto.5941 18376138

[31] Tsujioka T Miura Y Otsuki T Nishimura Y Hyodoh F Wada H . The mechanisms of vitamin K2-induced apoptosis of myeloma cells. Haematologica. (2006) 91:613–9. 16670066

[32] Martin M . Cutadapt removes adapter sequences from high-throughput sequencing reads. EMBnet J. (2011) 17:10–2. doi: 10.14806/ej.17.1.200

[33] Dobin A Davis CA Schlesinger F Drenkow J Zaleski C Jha S . STAR: ultrafast universal RNA-seq aligner. Bioinformatics. (2013) 29:15–20. doi: 10.1093/bioinformatics/bts635 23104886 PMC3530905

[34] Yates AD Achuthan P Akanni W Allen J Allen J Alvarez-Jarreta J . Ensembl 2020. Nucleic Acids Res. (2020) 48:D682–8. doi: 10.1093/nar/gkz966 31691826 PMC7145704

[35] Liao Y Smyth GK Shi W . featureCounts: an efficient general purpose program for assigning sequence reads to genomic features. Bioinformatics. (2014) 30:923–30. doi: 10.1093/bioinformatics/btt656 24227677

[36] Aryee MJ Jaffe AE Corrada-Bravo H Ladd-Acosta C Feinberg AP Hansen KD . Minfi: a flexible and comprehensive Bioconductor package for the analysis of Infinium DNA methylation microarrays. Bioinformatics. (2014) 30:1363–9. doi: 10.1093/bioinformatics/btu049 24478339 PMC4016708

[37] Fortin J-P Triche TJ Hansen KD . Preprocessing, normalization and integration of the Illumina HumanMethylationEPIC array with minfi. Bioinformatics. (2017) 33:558–60. doi: 10.1093/bioinformatics/btw691 28035024 PMC5408810

[38] Triche TJ Weisenberger DJ Van Den Berg D Laird PW Siegmund KD . Low-level processing of illumina infinium DNA methylation beadArrays. Nucleic Acids Res. (2013) 41:e90. doi: 10.1093/nar/gkt090 23476028 PMC3627582

[39] Morris TJ Butcher LM Feber A Teschendorff AE Chakravarthy AR Wojdacz TK . ChAMP: 450k chip analysis methylation pipeline. Bioinformatics. (2014) 30:428–30. doi: 10.1093/bioinformatics/btt684 24336642 PMC3904520

[40] Johnson WE Li C Rabinovic A . Adjusting batch effects in microarray expression data using empirical Bayes methods. Biostatistics. (2007) 8:118–27. doi: 10.1093/biostatistics/kxj037 16632515

[41] Du P Zhang X Huang CC Jafari N Kibbe WA Hou L . Comparison of Beta-value and M-value methods for quantifying methylation levels by microarray analysis. BMC Bioinf. (2010) 11:587. doi: 10.1186/1471-2105-11-587 21118553 PMC3012676

[42] Rainer J Gatto L Weichenberger CX . ensembldb: an R package to create and use Ensembl-based annotation resources. Bioinformatics. (2019) 35:3151–3. doi: 10.1093/bioinformatics/btz031 30689724 PMC6736197

[43] Yu G Wang LG Han Y He QY . clusterProfiler: an R package for comparing biological themes among gene clusters. OMICS. (2012) 16:284–7. doi: 10.1089/omi.2011.0118 22455463 PMC3339379

[44] Wilkinson L . ggplot2: Elegant graphics for data analysis by Wickham H. Biometrics. (2011) 67:671–9. doi: 10.1111/j.1541-0420.2011.01616.x40046247

[45] Anwar S Haq F Saeed M . PFKP mediates breast cancer metastasis through altered glycolysis. Cureus. (2025) 17:e87880. doi: 10.7759/cureus.87880 40821334 PMC12351505

[46] Shen J Jin Z Lv H Jin K Jonas K Zhu C . PFKP is highly expressed in lung cancer and regulates glucose metabolism. Cell Oncol (Dordr). (2020) 43:617–29. doi: 10.1007/s13402-020-00508-6 32219704 PMC12990724

[47] Lu TJ Yang YF Cheng CF Tu YT Chen YR Lee MC . Phosphofructokinase platelet overexpression accelerated colorectal cancer cell growth and motility. J Cancer. (2023) 14:943–51. doi: 10.7150/jca.82738 37151384 PMC10158518

[48] Liu B Li F Liu M Xu Z Gao B Wang Y . Prognostic roles of phosphofructokinase platelet in clear cell renal cell carcinoma and correlation with immune infiltration. Int J Gen Med. (2021) 14:3645–58. doi: 10.2147/IJGM.S321337 34321910 PMC8312753

[49] Rothhammer T Poser I Soncin F Bataille F Moser M Bosserhoff AK . Bone morphogenic proteins are overexpressed in Malignant melanoma and promote cell invasion and migration. Cancer Res. (2005) 65:448–56. doi: 10.1158/0008-5472.448.65.2 15695386

[50] Bao Z Zhang C Yan W Liu Y Li M Zhang W . BMP4, a strong better prognosis predictor, has a subtype preference and cell development association in gliomas. J Transl Med. (2013) 11:100. doi: 10.1186/1479-5876-11-100 23590708 PMC3637580

[51] He H Zhang J Qu Y Wang Y Zhang Y Yan X . Novel tumor-suppressor FOXN3 is downregulated in adult acute myeloid leukemia. Oncol Lett. (2019) 18:1521–9. doi: 10.3892/ol.2019.10424 31423219 PMC6607153

[52] Sun J Li H Huo Q Cui M Ge C Zhao F . The transcription factor FOXN3 inhibits cell proliferation by downregulating E2F5 expression in hepatocellular carcinoma cells. Oncotarget. (2016) 7:43534–45. doi: 10.18632/oncotarget.9780 27259277 PMC5190042

[53] Massey S Khan MA Rab SO Husain SM Khan AS Mallik Z . Clinical significance of FOXN3 expression in Indian breast cancer patients. Sci Rep. (2025) 15:13414. doi: 10.1038/s41598-025-98090-0 40251258 PMC12008265

[54] Joseph J Radulovich N Wang T Raghavan V Zhu CQ Tsao MS . Rho guanine nucleotide exchange factor ARHGEF10 is a putative tumor suppressor in pancreatic ductal adenocarcinoma. Oncogene. (2020) 39:308–21. doi: 10.1038/s41388-019-0985-1 31477830

[55] Bloedjes TA de Wilde G Khan GH Ashby TC Shaughnessy JD Zhan F . AKT supports the metabolic fitness of multiple myeloma cells by restricting FOXO activity. Blood Adv. (2023) 7:1697–712. doi: 10.1182/bloodadvances.2022007383 36322819 PMC10182179

[56] Farhan M Silva M Xingan X Huang Y Zheng W . Role of FOXO transcription factors in cancer metabolism and angiogenesis. Cells. (2020) 9:1586. doi: 10.3390/cells9071586 32629884 PMC7407656

[57] Austen B Podinovskaia M Starcynski J Barone G Reiman A Byrd P . Identification of ATM mutations in multiple myeloma tumours. Blood. (2005) 106:1562. doi: 10.1182/blood.V106.11.1562.1562

[58] Wu P Wen Z . ATM is associated with the prognosis of colorectal cancer: a systematic review. Front Oncol. (2025) 15:1470939. doi: 10.3389/fonc.2025.1470939 40144209 PMC11936800

[59] He X Ma Y Huang Z Wang G Wang W Zhang R . SERPINB5 is a prognostic biomarker and promotes proliferation, metastasis and epithelial-mesenchymal transition (EMT) in lung adenocarcinoma. Thorac Cancer. (2023) 14:2275–87. doi: 10.1111/1759-7714.15013 37424293 PMC10423661

[60] Liu BX Xie Y Zhang J Zeng S Li J Tao Q . SERPINB5 promotes colorectal cancer invasion and migration by promoting EMT and angiogenesis via the TNF-α/NF-κB pathway. Int Immunopharmacol. (2024) 131:111759. doi: 10.1016/j.intimp.2024.111759 38460302

[61] Shi S Zhang ZG Sang YZ Sun J Ma HY . A meta- and bioinformatics analysis of maspin expression levels influencing the prognosis of patients with breast cancer. Oncol Lett. (2024) 27:173. doi: 10.3892/ol.2024.14306 38464336 PMC10921733

[62] Shreenivas A Janku F Gouda MA Chen HZ George B Kato S . ALK fusions in the pan-cancer setting: another tumor-agnostic target? NPJ Precis Onc. (2023) 7:101. doi: 10.1038/s41698-023-00449-x 37773318 PMC10542332

[63] Donzelli S Milano E Pruszko M Sacconi A Masciarelli S Iosue I . Expression of ID4 protein in breast cancer cells induces reprogramming of tumour-associated macrophages. Breast Cancer Res. (2018) 20:59. doi: 10.1186/s13058-018-0990-2 29921315 PMC6009061

[64] Ha C-T Cheng C-Y Zheng M-Y Hsu T-H Miao C-C Lee C-J . ID4 predicts poor prognosis and promotes BDNF-mediated oncogenesis of colorectal cancer. Carcinogenesis. (2021) 42:951–60. doi: 10.1093/carcin/bgab037 33993270

[65] Kawasaki T Yokoi S Tsuda H Izumi H Kozaki K Aida S . BCL2L2 is a probable target for novel 14q11.2 amplification detected in a non-small cell lung cancer cell line. Cancer Sci. (2007) 98:1070–7. doi: 10.1111/j.1349-7006.2007.00491.x 17459056 PMC11159300

[66] Lyu Y Li K Li Y Wen H Feng C . BCL2L2 loss renders -14q renal cancer dependent on BCL2L1 that mediates resistance to tyrosine kinase inhibitors. Clin Transl Med. (2021) 11:e348. doi: 10.1002/ctm2.348 33784008 PMC7933017

[67] Qiu S Lin W Zhou Z Hong Q Chen S Li J . TOX: a potential new immune checkpoint in cancers by pancancer analysis. Discov Oncol. (2024) 15:354. doi: 10.1007/s12672-024-01236-1 39152366 PMC11329495

[68] Zhao Y Liao P Huang S Deng T Tan J Huang Y . Increased TOX expression associates with exhausted T cells in patients with multiple myeloma. Exp Hematol Oncol. (2022) 11:12. doi: 10.1186/s40164-022-00267-0 35246241 PMC8895562

[69] Hayashi M Okabe K Yamawaki Y Teranishi M Honoki K Mori T . Loss of lysophosphatidic acid receptor-3 enhances cell migration in rat lung tumor cells. Biochem Biophys Res Commun. (2011) 405:450–4. doi: 10.1016/j.bbrc.2011.01.051 21255556

[70] Su P Xiao L Ye L Wang Z Xiong W Wang Q . A novel role of lysophosphatidic acid (LPA) in human myeloma resistance to proteasome inhibitors. J Hematol Oncol. (2022) 15:55. doi: 10.1186/s13045-022-01269-5 35526043 PMC9077919

[71] Kanehira M Fujiwara T Nakajima S Okitsu Y Onishi Y Fukuhara N . An Lysophosphatidic Acid Receptors 1 and 3 axis governs cellular senescence of mesenchymal stromal cells and promotes growth and vascularization of multiple myeloma. Stem Cells. (2017) 35:739–53. doi: 10.1002/stem.2499 27641212

[72] Singh I Karna A Prajapati A Solanki U Mukherjee A Uppal S . Epigenetic targeting of MECOM/KRAS axis by JIB-04 impairs tumorigenesis and cisplatin resistance in MECOM-amplified ovarian cancer. Cell Death Discov. (2025) 11:326. doi: 10.1038/s41420-025-02618-2 40664642 PMC12264112

[73] Lou M Zou L Zhang L Lu Y Chen J Zong B . MECOM and the PRDM gene family in uterine endometrial cancer: bioinformatics and experimental insights into pathogenesis and therapeutic potentials. Mol Med. (2024) 30:190. doi: 10.1186/s10020-024-00946-0 39468462 PMC11514642

[74] Polprasert C Chanswangphuwana C Owattanapanich W Kungwankiattichai S Rattarittamrong E Rattanathammethee T . Clinical characteristics and outcomes of myeloid neoplasms with MECOM rearrangement: results from a nationwide multicenter study. Blood. (2023) 142:4213. doi: 10.1182/blood-2023-180100

[75] Kątnik E Gomułkiewicz A Piotrowska A Grzegrzółka J Rusak A Kmiecik A . BCL11A expression in breast cancer. Curr Issues Mol Biol. (2023) 45:2681–98. doi: 10.3390/cimb45040175 37185699 PMC10137054

[76] Tao H Ma X Su G Yin J Xie X Hu C . BCL11A expression in acute myeloid leukemia. Leuk Res. (2016) 41:71–5. doi: 10.1016/j.leukres.2015.12.001 26707798

[77] Yin J Xie X Ye Y Wang L Che F . BCL11A: a potential diagnostic biomarker and therapeutic target in human diseases. Biosci Rep. (2019) 39:BSR20190604. doi: 10.1042/BSR20190604 31654056 PMC6851505

[78] Zhang J Lv H Ji M Wang Z Wu W . Low circadian clock genes expression in cancers: a meta-analysis of its association with clinicopathological features and prognosis. PloS One. (2020) 15:e0233508. doi: 10.1371/journal.pone.0233508 32437452 PMC7241715

[79] Sanford ABA da Cunha LS MaChado CB de Pinho Pessoa FMC Silva ANDS Ribeiro RM . Circadian rhythm dysregulation and leukemia development: the role of clock genes as promising biomarkers. Int J Mol Sci. (2022) 23:8212. doi: 10.3390/ijms23158212 35897788 PMC9332415

[80] Gong X Tang H Yang K . PER1 suppresses glycolysis and cell proliferation in oral squamous cell carcinoma via the PER1/RACK1/PI3K signaling complex. Cell Death Dis. (2021) 12:276. doi: 10.1038/s41419-021-03563-5 33723221 PMC7960720

[81] Ma J Qin X Le W Chen X Wang X Xu C . Identification of BBC3 as a novel indicator for predicting prostate cancer development and olaparib resistance. Discov Oncol. (2024) 15:496. doi: 10.1007/s12672-024-01373-7 39331229 PMC11436583

[82] Hu J Van Valckenborgh E Xu D Menu E De Raeve H De Bruyne E . Synergistic induction of apoptosis in multiple myeloma cells by bortezomib and hypoxia-activated prodrug TH-302, *in vivo* and *in vitro*. Mol Cancer Ther. (2013) 12:1763–73. doi: 10.1158/1535-7163.MCT-13-0123 23832122

[83] Wang Q Weng S Sun Y Lin Y Zhong W Kwok HF . High DAPK1 expression promotes tumor metastasis of gastric cancer. Biol (Basel). (2022) 11:1488. doi: 10.3390/biology11101488 36290392 PMC9598723

[84] Zhao J Zhao D Poage GM Mazumdar A Zhang Y Hill JL . Death-associated protein kinase 1 promotes growth of p53-mutant cancers. J Clin Invest. (2015) 125:2707–20. doi: 10.1172/JCI70805 26075823 PMC4563671

[85] Song Z Li Z Han W Zhu C Lou N Li X . Low DAPK1 expression correlates with poor prognosis and sunitinib resistance in clear cell renal cell carcinoma. Aging (Albany NY). (2020) 13:1842–58. doi: 10.18632/aging.103638 33201837 PMC7880360

[86] Du F Dong D Zhang X Jia J . MXD1 is a potential prognostic biomarker and correlated with specific molecular change and tumor microenvironment feature in esophageal squamous cell carcinoma. Technol Cancer Res Treat. (2021) 20:15330338211052142. doi: 10.1177/15330338211052142 34761715 PMC8591776

[87] Raninga PV Di Trapani G Vuckovic S Tonissen KF . Targeted knockdown of DJ-1 induces multiple myeloma cell death via KLF6 upregulation. Apoptosis. (2016) 21:1422–37. doi: 10.1007/s10495-016-1303-9 27734217

[88] DiFeo A Martignetti JA Narla G . The role of KLF6 and its splice variants in cancer therapy. Drug Resist Update. (2009) 12:1–7. doi: 10.1016/j.drup.2008.11.001 19097929

[89] Cai D Zhong G-C Dai X Zhao Z Chen M Hu J . Targeting FDFT1 reduces cholesterol and bile acid production and delays hepatocellular carcinoma progression through the HNF4A/ALDOB/AKT1 axis. Adv Sci. (2025) 12:2411719. doi: 10.1002/advs.202411719 39899681 PMC11948044

[90] Ha NT Lee CH . Roles of farnesyl-diphosphate farnesyltransferase 1 in tumour and tumour microenvironments. Cells. (2020) 9:2352. doi: 10.3390/cells9112352 33113804 PMC7693003

[91] Chattopadhyay T Mallick B . FDFT1 repression by piR-39980 prevents oncogenesis by regulating proliferation and apoptosis through hypoxia in tongue squamous cell carcinoma. Life Sci. (2023) 329:121954. doi: 10.1016/j.lfs.2023.121954 37473805

[92] Calvert AE Chalastanis A Wu Y Hurley LA Kouri FM Bi Y . Cancer-associated IDH1 promotes growth and resistance to targeted therapies in the absence of mutation. Cell Rep. (2017) 19:1858–73. doi: 10.1016/j.celrep.2017.05.014 28564604 PMC5564207

[93] Liu M Li J Wang Y Ghaffar M Yang Y Wang M . MAGEA6 positively regulates MSMO1 and promotes the migration and invasion of oesophageal cancer cells. Exp Ther Med. (2022) 23:204. doi: 10.3892/etm.2022.11127 35126707 PMC8796618

[94] Zou J Liu S Long J . Up-regulation of MSMO1 was associated with poor survival in cervical cancer. Transl Cancer Res. (2024) 13:5316–27. doi: 10.21037/tcr-24-243 39525016 PMC11543037

[95] Pan H Zheng Y Pan Q Chen H Chen F Wu J . Expression of LXR-β, ABCA1 and ABCG1 in human triple-negative breast cancer tissues. Oncol Rep. (2019) 42:1869–77. doi: 10.3892/or.2019.7279 31432185 PMC6775801

[96] Viaud M Abdel-Wahab O Gall J Ivanov S Guinamard R Sore S . ABCA1 exerts tumor-suppressor function in myeloproliferative neoplasms. Cell Rep. (2020) 30:3397–3410.e5. doi: 10.1016/j.celrep.2020.02.056 32160545 PMC7473128

[97] Wang H Zhao S Liu H Liu Y Zhang Z Zhou Z . ALKBH5 facilitates the progression of skin cutaneous melanoma via mediating ABCA1 demethylation and modulating autophagy in an m^6^A-dependent manner. Int J Biol Sci. (2024) 20:1729–43. doi: 10.7150/ijbs.92994 38481816 PMC10929202

[98] Peng L Yang Z Tan C Ren G Chen J . Epigenetic inactivation of ADAMTS9 via promoter methylation in multiple myeloma. Mol Med Rep. (2013) 7:1055–61. doi: 10.3892/mmr.2013.1291 23358566

[99] Sun W Ma G Zhang L Wang P Zhang N Wu Z . DNMT3A-mediated silence in ADAMTS9 expression is restored by RNF180 to inhibit viability and motility in gastric cancer cells. Cell Death Dis. (2021) 12:428. doi: 10.1038/s41419-021-03628-5 33931579 PMC8087691

[100] Hu N Zou L Wang C Song G . RUNX1T1 function in cell fate. Stem Cell Res Ther. (2022) 13:369. doi: 10.1186/s13287-022-03074-w 35902872 PMC9330642

[101] Du J Guan M Fan J Jiang H . Loss of DMBT1 expression in human prostate cancer and its correlation with clinical progressive features. Urology. (2011) 77:509.e9–13. doi: 10.1016/j.urology.2010.09.023 21167560

[102] Wu W Kemp BL Proctor ML Gazdar AF Minna JD Hong WK . Expression of DMBT1, a candidate tumor suppressor gene, is frequently lost in lung cancer. Cancer Res. (1999) 59:1846–51. 10213490

[103] Yang W Wang B Yu Q Liu T Li T Tian T . ARHGAP24 represses β-catenin transactivation-induced invasiveness in hepatocellular carcinoma mainly by acting as a GTPase-independent scaffold. Theranostics. (2022) 12:6189–206. doi: 10.7150/thno.72134 36168627 PMC9475462

[104] Zhang S Sui L Zhuang J He S Song Y Ye Y . ARHGAP24 regulates cell ability and apoptosis of colorectal cancer cells via the regulation of P53. Oncol Lett. (2018) 16:3517–24. doi: 10.3892/ol.2018.9075 30127956 PMC6096278

[105] Wysocki P Kolanowska M Nowis D . 22P Functional characterization of DIRC3 long non-coding RNA in differentiated thyroid cancer. Ann Oncol. 32S367. doi: 10.1016/j.annonc.2021.08.30042574925

[106] Coe EA Tan JY Shapiro M Louphrasitthiphol P Bassett AR Marques AC . The MITF-SOX10 regulated long non-coding RNA DIRC3 is a melanoma tumour suppressor. PloS Genet. (2019) 15:e1008501. doi: 10.1371/journal.pgen.1008501 31881017 PMC6934268

[107] Parr C Jiang WG . Metastasis suppressor 1 (MTSS1) demonstrates prognostic value and anti-metastatic properties in breast cancer. Eur J Cancer. (2009) 45:1673–83. doi: 10.1016/j.ejca.2009.02.019 19328678

[108] Schemionek M Herrmann O Reher MM Chatain N Schubert C Costa IG . Mtss1 is a critical epigenetically regulated tumor suppressor in CML. Leukemia. (2016) 30:823–32. doi: 10.1038/leu.2015.329 26621336

[109] Tanaka T Ichimura K Sato Y Takata K Morito T Tamura M . Frequent downregulation or loss of CD79a expression in plasma cell myelomas: Potential clue for diagnosis. Pathol Int. (2009) 59:804–8. doi: 10.1111/j.1440-1827.2009.02448.x 19883431

[110] Lin YW Weng XF Huang BL Guo HP Xu YW Peng YH . IGFBP-1 in cancer: expression, molecular mechanisms, and potential clinical implications. Am J Transl Res. (2021) 13:813–32. PMC8014352 33841624

[111] Peng P Wu W Zhao J Song S Wang X Jia D . Decreased expression of Calpain-9 predicts unfavorable prognosis in patients with gastric cancer. Sci Rep. (2016) 6:29604. doi: 10.1038/srep29604 27404891 PMC4941732

[112] Palomer X Salvador JM Griñán-Ferré C Barroso E Pallàs M Vázquez-Carrera M . GADD45A: with or without you. Med Res Rev. (2024) 44:1375–403. doi: 10.1002/med.22015 38264852

[113] Hassan N Yi H Malik B Gaspard-Boulinc L Samaraweera SE Casolari DA . Loss of the stress sensor GADD45A promotes stem cell activity and ferroptosis resistance in LGR4/HOXA9-dependent AML. Blood. (2024) 144:84–98. doi: 10.1182/blood.2024024072 38579286 PMC11251412

[114] Mohamed-Hadley A Gibbs JD Liebermann DA Hoffman B . Gadd45a behaves as a tumor suppressor and gadd45b behaves as an oncogene in myc-mediated block in myeloid differentiation. Blood. (2007) 110:4163. doi: 10.1182/blood.V110.11.4163.4163

[115] You J Bian J Chen J Xia T Deng A Zhang M . TNFSF15 and MIA variant associated with immunotherapy and prognostic evaluation in esophageal cancer. J Oncol. (2023) 10:1248024. doi: 10.1155/2023/1248024 36936375 PMC10023233

[116] Lu HJ Chuang CY Su CW Chen MK Yang WE Yeh CM . Role of TNFSF15 variants in oral cancer development and clinicopathologic characteristics. J Cell Mol Med. (2022) 26:5452–62. doi: 10.1111/jcmm.17569 36226563 PMC9639028

[117] Hu L Chen HY Cai J Zhang Y Qi CY Gong H . Serine threonine tyrosine kinase 1 is a potential prognostic marker in colorectal cancer. BMC Cancer. (2015) 15:246. doi: 10.1186/s12885-015-1285-y 25884558 PMC4404069

[118] Orang AV Safaralizadeh R Hosseinpour Feizi MA Somi MH . Diagnostic relevance of overexpressed serine threonine tyrosine kinase/novel oncogene with kinase domain (STYK1/ NOK) mRNA in colorectal cancer. Asian Pac J Cancer Prev. (2014) 15:6685–9. doi: 10.7314/apjcp.2014.15.16.6685 25169509

[119] Hu YP Wu ZB Jiang L Jin YP Li HF Zhang YJ . STYK1 promotes cancer cell proliferation and Malignant transformation by activating PI3K-AKT pathway in gallbladder carcinoma. Int J Biochem Cell Biol. (2018) 97:16–27. doi: 10.1016/j.biocel.2018.01.016 29413947

[120] Giannoni P de Totero D . The HGF/c-MET axis as a potential target to overcome survival signals and improve therapeutic efficacy in multiple myeloma. Cancer Drug Resist. (2021) 4:923–33. doi: 10.20517/cdr.2021.73 35582373 PMC8992445

[121] Ueki T Fujimoto J Suzuki T Yamamoto H Okamoto E . Expression of hepatocyte growth factor and its receptor c-metproto-oncogene in hepatocellular carcinoma. Hepatology. (1997) 25:862–6. doi: 10.1002/hep.510250413 9096589

[122] Pidugu VK Wu MM Yen AH Pidugu HB Chang KW Liu CJ . IFIT1 and IFIT3 promote oral squamous cell carcinoma metastasis and contribute to the anti-tumor effect of gefitinib via enhancing p-EGFR recycling. Oncogene. (2019) 38:3232–47. doi: 10.1038/s41388-018-0662-9 30626937

[123] Zhao Y Zhang Y Lu W Sun R Guo R Cao X . The diagnostic/prognostic roles and biological function of the IFIT family members in acute myeloid leukemia. BMC Med Genomics. (2023) 16:296. doi: 10.1186/s12920-023-01735-0 37980495 PMC10657597

[124] Hendrix A Maynard D Pauwels P Braems G Denys H Van den Broecke R . Effect of the secretory small GTPase Rab27B on breast cancer growth, invasion, and metastasis. J Natl Cancer Inst. (2010) 102:866–80. doi: 10.1093/jnci/djq153 20484105 PMC2886092

[125] Ren P Yang XQ Zhai XL Zhang YQ Huang JF . Overexpression of Rab27B is correlated with distant metastasis and poor prognosis in ovarian cancer. Oncol Lett. (2016) 12:1539–45. doi: 10.3892/ol.2016.4801 27446467 PMC4950177

[126] Wei Y Chen X Ren X Wang B Zhang Q Bu H . Identification of MX2 as a novel prognostic biomarker for sunitinib resistance in clear cell renal cell carcinoma. Front Genet. (2021) 12:680369. doi: 10.3389/fgene.2021.680369 34306023 PMC8299280

[127] Kotov JA Xu Y Carey ND Cyster JG . LTβR overexpression promotes plasma cell accumulation. PloS One. (2022) 17:e0270907. doi: 10.1371/journal.pone.0270907 35925983 PMC9352096

[128] Wu Y Zhao S Guo W Liu Y Requena Mullor MDM Rodrìguez RA . Systematic analysis of the prognostic value and immunological function of LTBR in human cancer. Aging (Albany NY). (2024) 16:129–52. doi: 10.18632/aging.205356 38175686 PMC10817409

[129] Scarzello AJ Jiang Q Back T Dang H Hodge D Hanson C . LTβR signalling preferentially accelerates oncogenic AKT-initiated liver tumours. Gut. (2016) 65:1765–75. doi: 10.1136/gutjnl-2014-308810 26206664 PMC5036232

[130] Atamaniuk J Gleiss A Porpaczy E Kainz B Grunt TW Raderer M . Overexpression of G protein-coupled receptor 5D in the bone marrow is associated with poor prognosis in patients with multiple myeloma. Eur J Clin Invest. (2012) 42:953–60. doi: 10.1111/j.1365-2362.2012.02679.x 22591013

[131] Toma MI Erdmann K Diezel M Meinhardt M Zastrow S Fuessel S . Lack of ephrin receptor A1 is a favorable independent prognostic factor in clear cell renal cell carcinoma. PloS One. (2014) 9:e102262. doi: 10.1371/journal.pone.0102262 25025847 PMC4099180

[132] Herath N Doecke J Spanevello M . Epigenetic silencing of EphA1 expression in colorectal cancer is correlated with poor survival. Br J Cancer. (2009) 100:1095–102. doi: 10.1038/sj.bjc.6604970 19277044 PMC2670002

[133] Su H Karin M . Multifaceted collagen-DDR1 signaling in cancer. Trends Cell Biol. (2024) 34:406–15. doi: 10.1016/j.tcb.2023.08.003 37709651 PMC10927612

[134] Zhou D Ren K Wang J Ren H Yang W Wang W . Erythropoietin-producing hepatocellular A6 overexpression is a novel biomarker of poor prognosis in patients with breast cancer. Oncol Lett. (2018) 15:5257–63. doi: 10.3892/ol.2018.7919 29552165 PMC5840743

[135] Raja E Morikawa M Nishida J Tanabe R Takahashi K Seeherman HJ . Tyrosine kinase Eph receptor A6 sensitizes glioma-initiating cells towards bone morphogenetic protein-induced apoptosis. Cancer Sci. (2019) 110:3486–96. doi: 10.1111/cas.14187 31483918 PMC6825014

[136] Yamanaka M Kanda K Li NC Fukumori T Oka N Kanayama HO . Analysis of the gene expression of SPARC and its prognostic value for bladder cancer. J Urol. (2001) 166:2495–9. doi: 10.1016/S0022-5347(05)65623-6 11696817

[137] Fotiou D Katodritou E . From biology to clinical practice: the bone marrow microenvironment in multiple myeloma. J Clin Med. (2025) 14:327. doi: 10.3390/jcm14020327 39860333 PMC11765558

[138] Hu J Van Valckenborgh E Menu E De Bruyne E Vanderkerken K . Understanding the hypoxic niche of multiple myeloma: therapeutic implications and contributions of mouse models. Dis Model Mech. (2012) 5:763–71. doi: 10.1242/dmm.008961 23115205 PMC3484859

[139] DeLuca HF . Overview of general physiologic features and functions of vitamin D. Am J Clin Nutr. (2004) 80:1689S–96S. doi: 10.1093/ajcn/80.6.1689S 15585789

[140] Wang Y Zhu J DeLuca HF . Where is the vitamin D receptor? Arch Biochem Biophys. (2012) 523:123–33. doi: 10.1016/j.abb.2012.04.001 22503810

[141] Dormanen MC Bishop JE Hammond MW Okamura WH Nemere I Norman AW . Nonnuclear effects of the steroid hormone 1 alpha,25(OH)2-vitamin D3: analogs are able to functionally differentiate between nuclear and membrane receptors. Biochem Biophys Res Commun. (1994) 201:394–401. doi: 10.1006/bbrc.1994.1714 8198601

[142] Nemere I Dormanen MC Hammond MW Okamura WH Norman AW . Identification of a specific binding protein for 1 alpha,25-dihydroxyvitamin D3 in basal-lateral membranes of chick intestinal epithelium and relationship to transcaltachia. J Biol Chem. (1994) 269:23750–6. doi: 10.1016/S0021-9258(17)31579-X 8089147

[143] Hii CS Ferrante A . The non-genomic actions of vitamin D. Nutrients. (2016) 8:135. doi: 10.3390/nu8030135 26950144 PMC4808864

[144] Kulig P Rałowiec A Baumert B Machaliński B . The role of IL-17-related signaling in myelomagenesis, disease prognosis/progression, and therapeutic approach-a scoping review. Clin Exp Med. (2025) 25:194. doi: 10.1007/s10238-025-01728-6 40488820 PMC12149252

[145] Kulig P Łuczkowska K Machaliński B Baumert B . Deep hematologic response to RD treatment in patients with multiple myeloma is associated with overexpression of IL-17R in CD138+ plasma cells. Sci Rep. (2024) 14:23559. doi: 10.1038/s41598-024-74558-3 39384864 PMC11464892

[146] Busch L Mougiakakos D Büttner-Herold M Müller MJ Volmer DA Bach C . Lenalidomide enhances MOR202-dependent macrophage-mediated effector functions via the vitamin D pathway. Leukemia. (2018) 32:2445–58. doi: 10.1038/s41375-018-0114-0 29654274

[147] Graklanov V Popov V Raycheva R . Serum levels of vitamin D in patients with multiple myeloma. Folia Med (Plovdiv). (2020) 62:730–5. doi: 10.3897/folmed.62.e50321 33415920

[148] Eicher F Mansouri Taleghani B Schild C Bacher U Pabst T . Reduced survival after autologous stem cell transplantation in myeloma and lymphoma patients with low vitamin D serum levels. Hematological Oncol. (2020) 38:523–30. doi: 10.1002/hon.2774 32594534

[149] Raoufinejad K Shamshiri AR Pezeshki S Chahardouli B Hadjibabaie M Jahangard-Rafsanjani Z . Oral calcitriol in hematopoietic recovery and survival after autologous stem cell transplantation: a randomized clinical trial. Daru. (2019) 27:709–20. doi: 10.1007/s40199-019-00306-y 31713184 PMC6895337

[150] Yellapragada SV Fillmore NR Frolov A Zhou Y Dev P Yameen H . Vitamin D deficiency predicts for poor overall survival in white but not African American patients with multiple myeloma. Blood Adv. (2020) 4:1643–6. doi: 10.1182/bloodadvances.2019001411 32315398 PMC7189302

