# Supplementary material for: Combined vitamins D3 and K2 as potential modulators of multiple myeloma cell pathophysiology: transcriptomic and epigenetic effects under standard culture and bone marrow-like conditions
Source: Front Oncol. 2026 Sep 14;16:1940737. doi: 10.3389/fonc.2026.1940737 (PMC13616678; doi:10.3389/fonc.2026.1940737)
Supplement: Supplementary file 1 [file DataSheet1.zip › supplemental figures/supplemental figures/Figure 5S.pdf]

Gene	MM1S_Control_H	MM1S_DK_H	Fold change	U266_Control_H	U266_DK_H	Fold change
PER1	1115.97	2689.93	2.41	1522.61	3312.36	2.17
BBC3	266.84	594.69	2.22	235.98	481.94	2.04
DAPK1	1870.34	1559.27	0.83	1435.35	2423.92	1.68
MXD1	344.94	762.65	2.21	486.73	811.20	1.66
KLF6	10229.95	13606.08	1.33	9365.68	14494.54	1.54
FDFT1	2645.16	1272.22	-2.08	2450.72	1665.15	-1.47
IDH1	710.26	240.73	-2.95	658.52	401.54	-1.64
MSMO1	1798.00	609.41	-2.95	1535.53	901.13	-1.70

Supplementary Figure 5S. Expression levels of genes commonly differentially expressed in U266 and MM1S cells following combined vitamin D3 and vitamin K2 treatment. Expression levels in untreated control and vitamin-treated cells and the corresponding fold changes are presented for both cell lines.
