# Supplementary material for: Combined vitamins D3 and K2 as potential modulators of multiple myeloma cell pathophysiology: transcriptomic and epigenetic effects under standard culture and bone marrow-like conditions
Source: Front Oncol. 2026 Sep 14;16:1940737. doi: 10.3389/fonc.2026.1940737 (PMC13616678; doi:10.3389/fonc.2026.1940737)
Supplement: Supplementary file 1 [file DataSheet1.zip › supplemental figures/supplemental figures/Figure_S1.pdf]

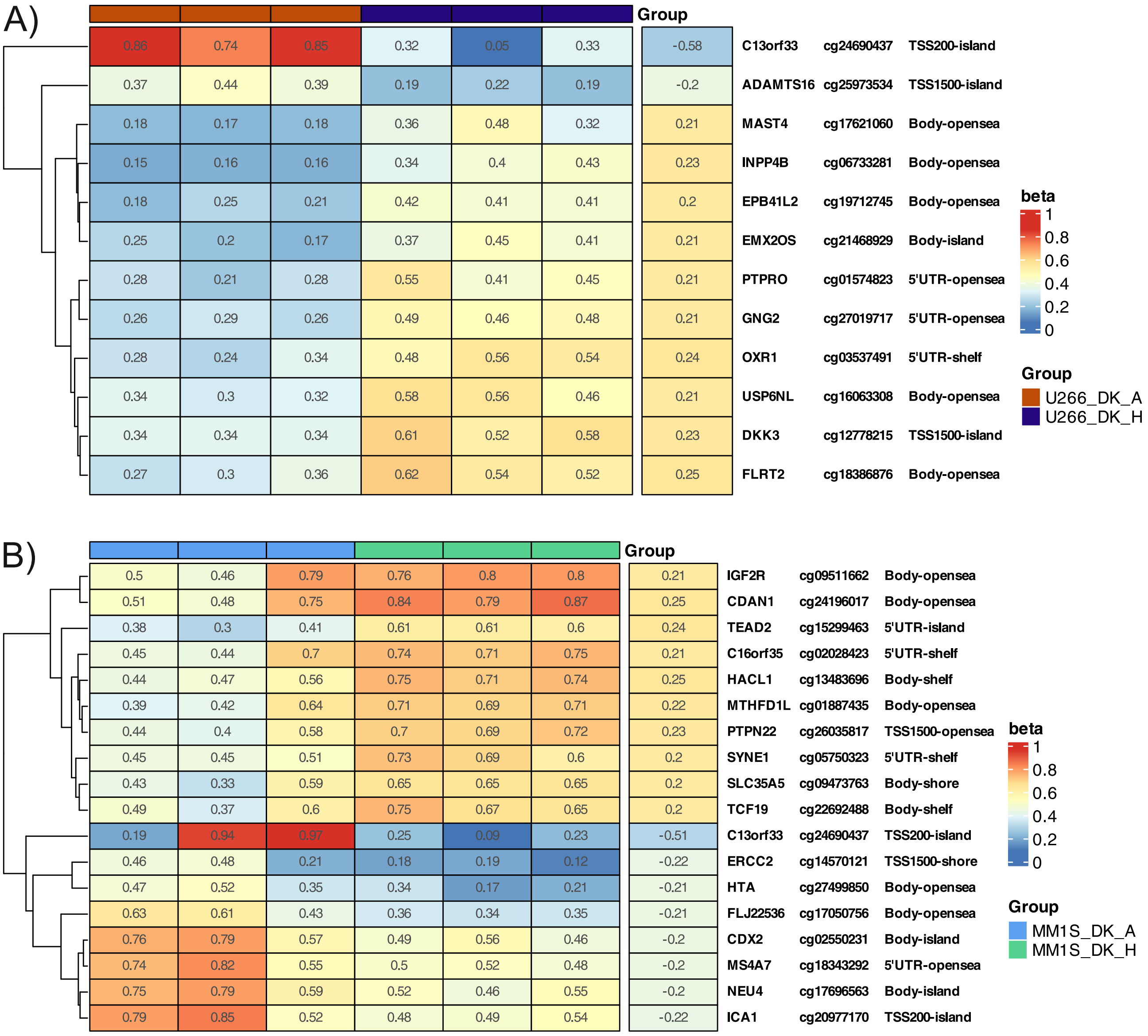

Figure S1. Heatmap showing DNA methylation changes in A) U266 and B) MM1S cell lines under hypoxic vs. normoxic conditions after vitamin incubation (DK). Rows represent genes and their beta values, and columns represent treatment conditions (H-hypoxic and A-normoxic/atmospheric conditions). Only genes with statistically significant methylation changes (padj < 0.05) are shown. Colors indicate the degree of methylation change, with blue representing hypomethylation and red representing hypermethylation. Data are based on genome-wide DNA methylation profiling, and genes were clustered using hierarchical clustering.
