# Supplementary material for: Combined vitamins D3 and K2 as potential modulators of multiple myeloma cell pathophysiology: transcriptomic and epigenetic effects under standard culture and bone marrow-like conditions
Source: Front Oncol. 2026 Sep 14;16:1940737. doi: 10.3389/fonc.2026.1940737 (PMC13616678; doi:10.3389/fonc.2026.1940737)
Supplement: Supplementary file 1 [file DataSheet1.zip › supplemental figures/supplemental figures/Figure_S2.pdf]

MM1S DK H vs. MM1S DK A

gene symbol	gene name	gene biotype	log2FC	p.adj
PFKFB4	6−phosphofructo−2−kinase/fructose−2,6−biphosphatase 4	protein_coding	2.90	3.1e−134
CA9	carbonic anhydrase 9	protein_coding	2.80	5.4e−10
MIR210HG	MIR210 host gene	lncRNA	2.50	2.0e−120
TNS1	tensin 1	protein_coding	2.10	1.4e−09
VLDLR	very low density lipoprotein receptor	protein_coding	1.90	2.2e−32
CDKN1C	cyclin dependent kinase inhibitor 1C	protein_coding	1.80	1.2e−35
AK4	adenylate kinase 4	protein_coding	1.70	1.8e−130
P4HA1	prolyl 4−hydroxylase subunit alpha 1	protein_coding	1.60	3.0e−252
PPFIA4	PTPRF interacting protein alpha 4	protein_coding	1.60	1.2e−117
TMEM45A	transmembrane protein 45A	protein_coding	1.50	5.7e−07
DLEC1	DLEC1 cilia and flagella associated protein	protein_coding	0.91	4.1e−02
CELSR3	cadherin EGF LAG seven−pass G−type receptor 3	protein_coding	0.74	3.3e−15
COL9A2	collagen type IX alpha 2 chain	protein_coding	0.66	4.7e−11
ALDH3B1	aldehyde dehydrogenase 3 family member B1	protein_coding	0.61	3.4e−10
RHBDF1	rhomboid 5 homolog 1	protein_coding	0.60	2.0e−02
TG	thyroglobulin	protein_coding	0.58	3.9e−02
VIM	vimentin	protein_coding	0.58	3.3e−30
RNASET2	ribonuclease T2	protein_coding	0.57	3.2e−03
SYNE2	spectrin repeat containing nuclear envelope protein 2	protein_coding	−0.51	6.7e−04
RIPOR3	RIPOR family member 3	protein_coding	−0.91	1.2e−03

Figure S2. Selected genes with the highest variability comparing MM1S cell line incubated with vitamins in hypoxic vs. normoxic conditions. (padj<0.05).
