# Supplementary material for: Combined vitamins D3 and K2 as potential modulators of multiple myeloma cell pathophysiology: transcriptomic and epigenetic effects under standard culture and bone marrow-like conditions
Source: Front Oncol. 2026 Sep 14;16:1940737. doi: 10.3389/fonc.2026.1940737 (PMC13616678; doi:10.3389/fonc.2026.1940737)
Supplement: Supplementary file 1 [file DataSheet1.zip › supplemental figures/supplemental figures/Figure_S3.pdf]

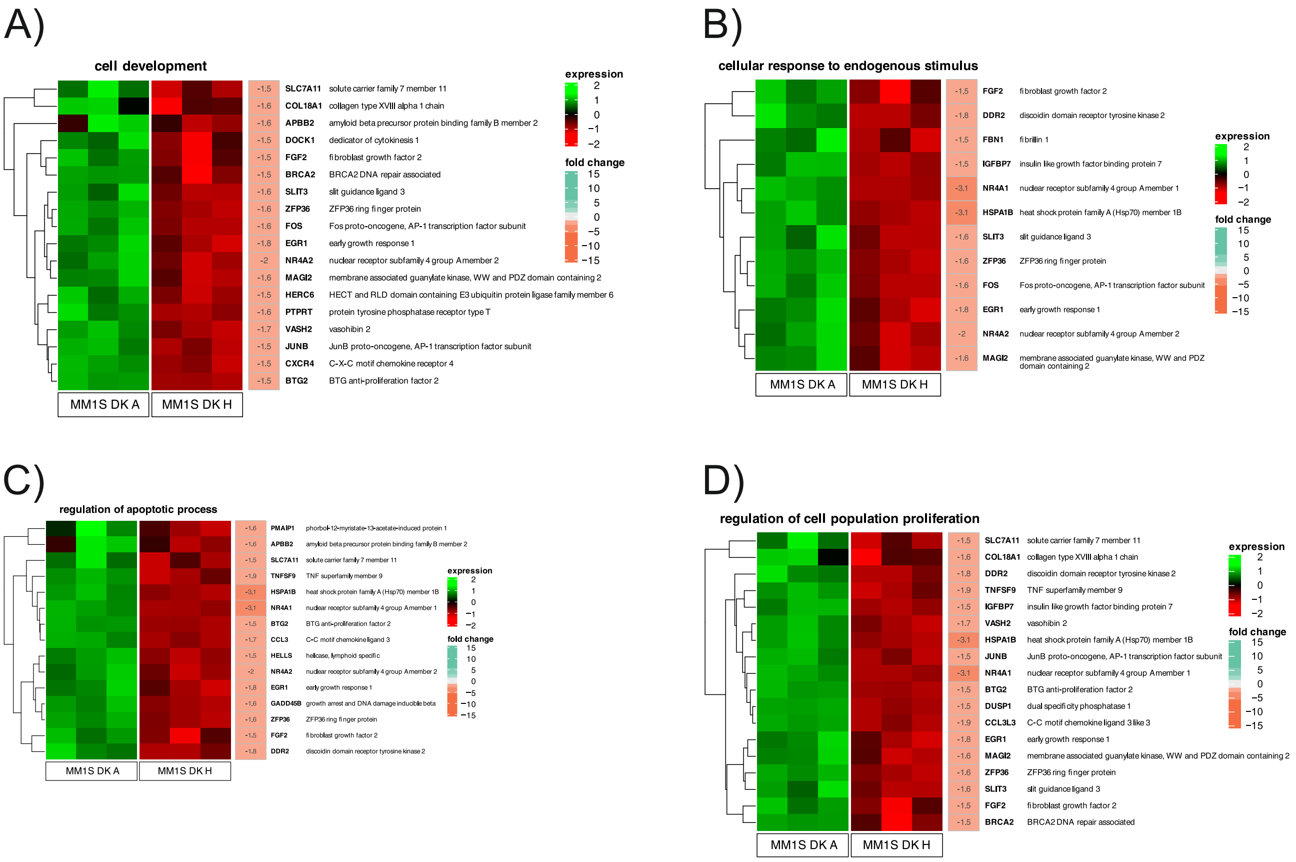

Figure S3. Heat maps for selected biological processes (a) cell development; b) cellular response to endogenous stimulus; c) regulation of apoptotic process; d) regulation of cell
